# Regional Patterning of Adult Neurogenesis in the Homing Pigeon’s Brain

**DOI:** 10.3389/fpsyg.2022.889001

**Published:** 2022-07-08

**Authors:** Julia Mehlhorn, Nelson Niski, Ke Liu, Svenja Caspers, Katrin Amunts, Christina Herold

**Affiliations:** ^1^Institute for Anatomy I, Medical Faculty and University Hospital Düsseldorf, Heinrich Heine University Düsseldorf, Düsseldorf, Germany; ^2^C. and O. Vogt-Institute for Brain Research, Medical Faculty and University Hospital Düsseldorf, Heinrich Heine University Düsseldorf, Düsseldorf, Germany; ^3^Institute of Neuroscience and Medicine (INM-1), Research Centre Jülich, Jülich, Germany

**Keywords:** adult neurogenesis, pigeon, avian brain, cell proliferation, plasticity, BrdU, NeuN, doublecortin

## Abstract

In the avian brain, adult neurogenesis has been reported in the telencephalon of several species, but the functional significance of this trait is still ambiguous. Homing pigeons (*Columba livia* f.d.) are well-known for their navigational skills. Their brains are functionally adapted to homing with, e.g., larger hippocampi. So far, no comprehensive mapping of adult neuro- and gliogenesis or studies of different developmental neuronal stages in the telencephalon of homing pigeons exists, although comprehensive analyses in various species surely will result in a higher understanding of the functional significance of adult neurogenesis. Here, adult, free flying homing pigeons were treated with 5-bromo-deoxyuridine (BrdU) to label adult newborn cells. Brains were dissected and immunohistochemically processed with several markers (GFAP, Sox2, S100ß, Tbr2, DCX, Prox1, Ki67, NeuN, Calbindin, Calretinin) to study different stages of adult neurogenesis in a quantitative and qualitative way. Therefore, immature and adult newborn neurons and glial cells were analyzed along the anterior–posterior axis. The analysis proved the existence of different neuronal maturation stages and showed that immature cells, migrating neurons and adult newborn neurons and glia were widely and regionally unequally distributed. Double- and triple-labelling with developmental markers allowed a stage classification of adult neurogenesis in the pigeon brain (1: continuity of stem cells/proliferation, 2: fate specification, 3: differentiation/maturation, 4: integration). The most adult newborn neurons and glia were found in the intercalated hyperpallium (HI) and the hippocampal formation (HF). The highest numbers of immature (DCX+) cells were detected in the nidopallium (N). Generally, the number of newborn glial cells exceeded the number of newborn neurons. Individual structures (e.g., HI, N, and HF) showed further variations along the anterior–posterior axis. Our qualitative classification and the distribution of maturing cells in the forebrain support the idea that there is a functional specialization, respectively, that there is a link between brain-structure and function, species-specific requirements and adult neurogenesis. The high number of immature neurons also suggests a high level of plasticity, which points to the ability for rapid adaption to environmental changes through additive mechanisms. Furthermore, we discuss a possible influence of adult neurogenesis on spatial cognition.

## Introduction

The generation, proliferation and maturation of new neurons in the brain over the lifespan (adult neurogenesis) have been detected in a variety of species including birds (e.g., Kirn and Nottebohm, 1993; Doetsch and Scharff, 2001; Lindsey and Tropepe, 2006; Balthazart et al., 2008; Kempermann, 2012; Melleu et al., 2013; Powers, 2016; Ngwenya et al., 2018).

In general, neurogenesis is a multistage process in which new neurons are born (division), develop (proliferation and maturation), move to specific brain regions (migration) and are then incorporated into existing brain circuits through dendritic growth and by forming synapses (differentiation; Barnea and Pravusodov, 2011). As during embryogenesis, adult neurogenesis occurs mainly in regions adjacent to the ventricles, in birds in the ventricular zone (VZ) and in mammals in the subventricular zone (SVZ) and the hippocampal subgranular zone (SGZ; Goldman and Nottebohm, 1983; Alvarez-Buylla et al., 1998; Garcia-Verdugo et al., 1998). Precursor cells in these germinal regions divide and migrate to their ultimate destinations where they differentiate into mature neurons of distinct phenotypes.

Adult neurogenesis was often considered as an archaic trait that was gradually lost during the course of evolution (Lindsey and Tropepe, 2006; Kaslin et al., 2008). The opinion was that natural selection favoured neuronal networks with fixed numbers of neurons and that “complex” brains require stable conditions and cannot cope with persistent new neurons and high levels of plasticity. This was in line with studies that showed high rates of adult neurogenesis and several neurogenic niches in the brains of, e.g., fishes, reptiles or birds, and low rates and few neurogenic niches in mammals including humans (Font et al., 2001; Zupanc, 2001; Balthazart et al., 2008; Kaslin et al., 2008; Melleu et al., 2013; Powers, 2016; Ngwenya et al., 2018). Generally, the function of adult neurogenesis may vary among taxa and likely within them, and surely depends on functionally involved brain regions. Up to date, it is not clear, how particular needs and/or evolutionary traces have directed the function of adult neurogenesis. The knowledge about the influence of adult neurogenesis on brain function is sparse, sometimes inconsistent and best studied for adult hippocampal neurogenesis in mammalian brains (for review see Barker et al., 2011; Denoth-Lippuner and Jessberger, 2021; Leal-Galicia et al., 2021). For adult hippocampal neurogenesis in mammals, Kempermann (2008) formulated a “neurogenic reserve hypothesis” what means that newborn neurons can act as a neurogenic reserve. Neuronal cell production early in life seems to be more important to enhanced hippocampal function later in life because its potential for generating new neurons with particular acute function (according to environmental demand). Adult hippocampal neurogenesis might provide the potential to remain flexible and plastic during learning when the individual is exposed to novelty and complexity, although the different functions and regions of the hippocampus have to be considered separately. Possibly, adult neurogenesis in general helps to adapt to environmental changes in an appropriate and rapid way (Kempermann, 2008, 2012; Johnson et al., 2010; Denoth-Lippuner and Jessberger, 2021). Nevertheless, there is still a lot of debate about the functional role of adult neurogenesis and the high heterogeneity in vertebrates, even among mammals, which does not make things easier (Bonfanti, 2016; Lipp and Bonfanti, 2016).

Although evidence of ongoing adult neurogenesis outside of the mentioned neurogenic regions is controversial, the incorporation of newborn neurons in the hippocampus (particularly in the dentate gyrus) and the olfactory bulb is widely confirmed in nearly all mammals apart from cetaceans (whales, dolphins, and porpoises) and some bat species (Altman, 1969; Eriksson et al., 1998; Gould et al., 1999a,b; Amrein et al., 2007; Johnson et al., 2010; Chawana et al., 2013; Patzke et al., 2015; Hevner, 2016). Ernst et al. (2014) and Bergmann et al. (2015) described adult neurogenesis also in the striatum of humans what seems to be unique among mammals. In the other classes of vertebrates (fishes, reptiles, amphibians, and birds) adult neurogenesis is more widespread and often occurs in several brain regions (Font et al., 2001; Zupanc, 2001; Balthazart et al., 2008; Kaslin et al., 2008; Melleu et al., 2013; Powers, 2016; Ngwenya et al., 2018). However, in the past those vertebrate classes have hardly been used as model systems in this context. Meanwhile, there was a paradigm change towards the notion that comprehensive analyses of adult neurogenesis in various vertebrate and invertebrate species will lead to a more complete understanding of the fundamental biology and evolution of adult neurogenesis (Lindsey and Tropepe, 2006; Powers, 2016; Ngwenya et al., 2018; Herold et al., 2019). Besides, such comprehensive analyses provide a better framework to test hypotheses about the functional significance of this trait.

In birds, adult neurogenesis was detected in a variety of avian species and it is known that new neurons are not limited to a few brain regions and are added throughout most of the avian telencephalon (Balthazart et al., 2008; Barnea and Pravusodov, 2011). The higher level of adult neurogenesis in birds suggests that ongoing neurogenesis, in contrast to mammals, is a common phenomenon in this vertebrate class and that this form of plasticity seems to be a fundamental feature of the avian forebrain (Alvarez-Buylla et al., 1994). Thus, adult neurogenesis in birds is well suited for experimental manipulations and investigations and birds are a valuable and more than appropriate model to investigate adult neurogenesis (Brenowitz and Larson, 2015). Here, the first evidence of adult neurogenesis in the avian brain came from Nottebohm and colleagues who published a series of studies showing that new cells are added to the “high vocal centre” (HVC) of adult canaries (Goldman and Nottebohm, 1983). They reported seasonal and hormonal changes in the volume of song-related nuclei, influenced by sex, sexual maturity, song complexity, species and testosterone level (Nottebohm, 2005). Further, song behaviour is regulated by a network of pallial and striatal nuclei and the song-control system shows extensive plasticity in adults, including ongoing neurogenesis in several brain nuclei (Nottebohm, 2004; Brenowitz and Larson, 2015). Those studies in songbirds suggest that adult neurogenesis is related to song learning.

Additional studies showed adult neurogenesis in the hippocampus, apical hyperpallium and mesopallium of food-storing birds (e.g., marsh tits or black-capped chickadees) and, at least in the hippocampus, also a relationship between cell proliferation, food-storing behaviour and spatial learning (Hall et al., 2014). Besides, parameters like experience (Patel et al., 1997), seasonality (Hoshooley et al., 2007; Sherry and MacDougall-Shackleton, 2015), age (Ling et al., 1997; Meskenaite et al., 2016), food-deprivation (Robertson et al., 2017) or environmental enrichment (Melleu et al., 2016) are related to adult neurogenesis in birds. But even though there has been some progress in this field, only little is known about the identity and function of the produced cells. The best-known area is still the HVC in songbirds where both interneurons and long projecting neurons are continuously incorporated during adulthood (Kirn and Nottebohm, 1993; Alvarez-Buylla et al., 1998; Doetsch and Scharff, 2001). Furthermore, it would be of particular interest to know to what extent adult neurogenesis affects cognitive functions. It seems to be that at least adult hippocampal neurogenesis in mammals has a functional relevance for the cognitive functions of the hippocampus (for review see Toda and Gage, 2018). However, it has to be considered that there is still contradictions in this aspect and that comparative views of adult neurogenesis in the SGZ indicate that its occurrence is highly variable, species specific, without recognizable common functions and sometimes even lacking (Lipp and Bonfanti, 2016).

One of the most popular animal models to study spatial learning in cognitive neurosciences is pigeons. Because of their excellent homing abilities, they were often used for research on navigation or orientation behavior (for review see Herold et al., 2015). Thereby the pigeon brain is well adapted to homing with, e.g., relatively enlarged hippocampi (Rehkämper et al., 2008), with the volume of the hippocampus being altered by navigational experience (Cnotka et al., 2008). Melleu et al. (2013) and Mazengenya et al. (2017) already provided a qualitative analysis about the distribution of proliferating cells and fibres in many prosencephalic regions in pigeons and suggested regional variations. Besides, Herold et al. (2019) has given a detailed insight on adult neurogenesis of the different hippocampal subdivisions, including the description and quantitative distribution of different stages of cell proliferation. Different levels of adult neurogenesis were observed in the hippocampal subdivisions as well as a functional specialization along the anterior–posterior axis. But, apart from the hippocampal formation (Herold et al., 2019), there is no quantitative and comprehensive overview about adult neuro- and gliogenesis in the (homing) pigeon *per se* or about different stages of cell maturation and proliferation and their spatial distribution in the telencephalon of birds. Besides, up to date there are only a few studies about the occurrence of adult neurogenesis regarding anterior and posterior variations. Therefore, here we would like to provide an advanced and comprehensive mapping of adult neurogenesis in the forebrain of the domestic pigeon. This will include nearly all pallial brain areas of the forebrain and different stages of cell proliferation. Additionally, we will present an analysis of maturing and newborn cells along the anterior–posterior axis of the pigeon forebrain.

With this study, we will receive for the first time a stage classification of different cell types during adult neurogenesis in the pigeon brain. Additionally, we will get a map of the pigeon forebrain for regions (a) with increased proliferation, (b) adult neurogenesis, (c) adult gliogenesis and (d) with high expression of markers for differentiation. Our results may reveal the regions of the homing pigeon brain with high demands on functional plasticity and will provide a fundamental basis for future studies. This may facilitate the interpretation and comparison of studies of adult neurogenesis between species. The observations outline a new understanding of brain plasticity, which will help to seek explanations for the adaptive significance of adult neurogenesis in birds, where and why it occurs, which factors may influence it and its importance for spatial learning in birds.

## Materials and Methods

### Animals

Nine homing pigeons (*Columba livia* f.d., 4 males and 5 females) originating from our local breeding stock were selected as subjects for the quantitative analysis. Additionally, four pigeons were used for the qualitative analysis of developmental stage markers. All pigeons hatched in February 2014, BrdU injections (see below) took place in February 2015. Perfusion of all animals was performed 3 months later in May. During their lifetime, pigeons were allowed to leave the loft to fly around several hours per day, but do not have separate release experience. Food and water were available *ad libitum*.

All applicable international, national, and/or institutional guidelines for the care and use of animals were followed. The study was approved by the Committee on the Ethics of Animal Experiments of the state of North Rhine-Westphalia, Germany (Ref. 84-02.04.2014.A345).

### Immunohistochemistry

For immunohistochemistry all pigeons were injected i.m. on three consecutive days with 5-bromo-desoxyuridine (BrdU, 50 mg/kg), a thymidine analogue that is inserted into the DNA in actively dividing cells after administration. BrdU labeling allows stereological estimation of the total number of cells as well as the simultaneous use of cell-type specific markers for the immunohistochemical identification of newly generated cells (Nowakowski et al., 1989). Twelve weeks after injection animals were deeply anesthetized with Pentobarbital (70 mg/kg) and subsequently perfused with saline solution and 4% paraformaldehyde. All brains were removed and postfixed overnight in 4% paraformaldehyde +30% sucrose. After at least 24 h cryoprotection (30% sucrose in phosphate buffer), the brains were frozen and completely serially sectioned (40 μm) in a coronal plane.

For the comprehensive overview of different types of cells and further stages during adult neuro-and gliogenesis, we processed at first a qualitative analysis. For this, the developmental markers Sox2 (anti-Sox2; 1:200; ab97959 abcam), GFAP (anti-GFAP, 1:5000, ab16997 abcam, United States), Tbr2 (anti-Tbr2; 1:200; AB15894 milipore), S100ß (anti-S100ß; 1:200; ab52642 abcam), Prox1 (anti-Prox1; 1:500; AB5475 milipore), Ki67 (anti-Ki67; 1:200; ab15580 abcam), Calretinin (CR; anti-Calretinin; 1:500; ABIN1742427, antibody-online), Calbindin (CB; anti-Calbindin; 1:1000; CB-38a, Swant, USA) and Doublecortin (anti-DCX; 1:500; sc8066, Santa Cruz) were used next to BrdU (anti-BrdU, 1:200, OBT0030, Abd serotec, United States). As secondary antibodies we used anti-mouse anti-rabbit, anti-chicken, anti-guinea pig and anti-goat in different combinations conjugated to either Alexa 647, CY3 or Alexa488 (all 1:200, Dianova, Germany). GFAP and Sox2 were used as markers for multipotential neural stem cells. GFAP was also used as a marker for glial cells at later stages, just like S100ß. Tbr2, Prox1 and DCX were used as markers for intermediate neuronal progenitor cells. DCX was used for both, the qualitative and quantitative analysis (see below). DCX is a microtubule-associated protein which is expressed in many advanced precursor cells or at an early post-mitotic stage of immature neurons and has been widely accepted as an indicator for adult neurogenesis, neuronal plasticity and provides an alternative to BrdU labelling (Brown et al., 2003; Balthazart and Ball, 2014). Compared to Tbr2 and Prox1, DCX was expressed subsequently during the maturation of adult newborn neurons (Knoth et al., 2010; Lavado et al., 2010; Kempermann et al., 2015; Denoth-Lippuner and Jessberger, 2021). CB and CR were used as markers that indicate late integrative phases of adult neurons. Besides, they are common markers for neurons that are typically bound to established neuronal networks in birds and mammals (Baimbridge et al., 1992; Hof et al., 1999; Heyers et al., 2008; Bruce et al., 2016). Ki67, a nuclear protein, is a proliferative marker and was expressed in dividing cells for the entire duration of their mitotic process (Kee et al., 2002). Additionally, we used Hoechst-33,342 (ThermoFisher Scientific, Germany) to visualize cell nuclei.

To specify the different cell types that express different fate and stage markers and to classify different stages for the pigeon brain, we processed double- and triple-staining. GFAP, S100ß, DCX and NeuN co-staining with BrdU was processed to show whether stem cells actively divide and whether new stem cells, glial cells or neurons were newly generated in the adult pigeon forebrain. Co-labelling of Sox2 and GFAP would demonstrate actively dividing stem cells at an early stage. GFAP and Sox2 co-staining was used to identify highly active zones of dividing stem cells. Adult gliogenesis was inspected with GFAP and S100ß staining in combination with BrdU labelling. Double- and triple-labelling of DCX, CB and CR should confirm whether these markers can be considered as markers of late integrative phases of adult neurons. To underpin the classification of different stages, Ki67 and DCX double labelling was processed.

Detailed processing was identical as described in Herold et al., 2019. Free-floating sections were rinsed 2 times in phosphate buffered saline (PBS) followed by incubating in 2 N HCL at 45°C for 30 min and 0.1 M borate buffer at pH 8.5 for 10 min. After this, sections were washed in PBS for 10 min followed by 3% goat serum (Vector, United States) in PBS + Triton-X 0.3% (PBS-T) for 60 min. Incubation with primary antibody mix processed overnight at 4°C. After that, sections were washed 3 times in PBS and then incubated with the secondary antibody mix (see above) for 2 h at darkness. For all further steps, a minimum of light or complete darkness was necessary. Again, sections were washed 3 times with PBS and 2 times with PB and then mounted on gelatin-coated glass slides followed by air-drying for 24 h. At last, slides were dipped into distilled water and directly cover-slipped with Fluoromount-G (Southern Biotec, United States).

For quantitative analyses, one series (consisting of about 25 sections), out of 10 series of the whole pigeon brain, was immunohistochemically processed for fluorescent double-labelling detection of doublecortin (DCX, anti-DCX, 1:500, ab18723, abcam, United States) and BrdU (see above). Each of these sections was analysed/quantified (see below). As secondary antibodies we used a mix containing goat anti-rat Alexa 488 (1:200) and goat anti-rabbit CY3 (1:500, both Jackson-Immuno Research, United States). A second series was processed for fluorescent triple-labelling detection of BrdU (see above), NeuN (anti-NeuN, 1:1000, MAB377, Millipore, Germany) and GFAP (anti-GFAP, 1:5000, ab16997 abcam, United States). Here, used secondary antibodies were donkey anti-mouse Alexa 647 (1:200, Dianova, Germany), goat anti-rabbit FITC (1:200, Cayman Chemical, United States) and goat anti-rat CY3 (1:200, Millipore, Germany). NeuN and GFAP served as markers for mature neurons and glial cells. Accordingly, newborn neurons were proved with BrdU/NeuN double-labelling and newborn glial cells with BrdU/GFAP double-labelling.

All further steps were identical to the labelling procedure as described above.

All antibodies were validated by control rounds without the primary or the secondary antibody, western blotting procedures or confirmed specific binding in previous studies (Supplementary Figure S1; Herold et al., 2019).

### Data Analysis

**Figure 1:**
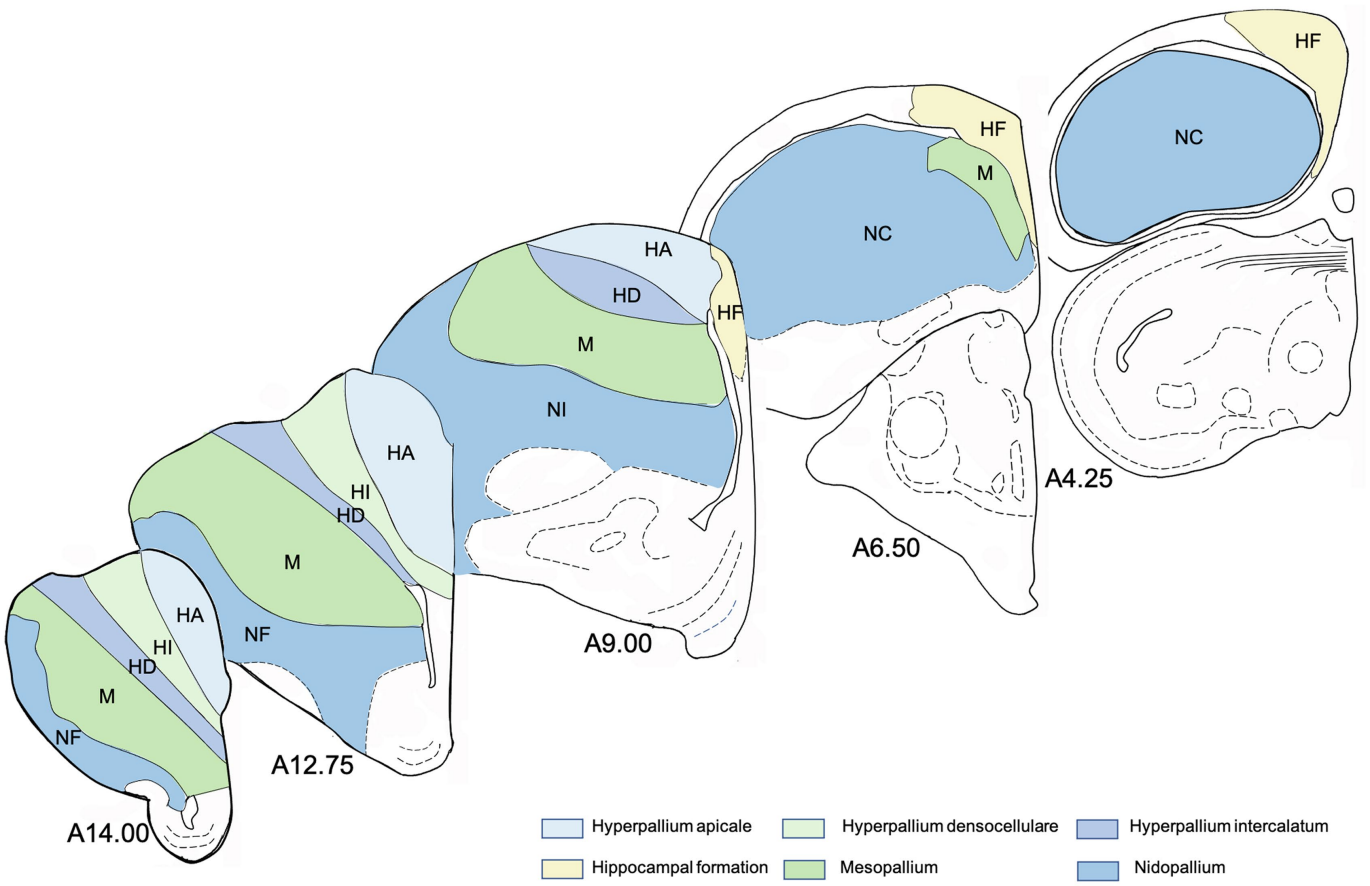
Schematic drawings of representative coronal sections of pigeons’ brain left hemisphere, from anterior to posterior levels about analysed regions in the brain of the pigeon. Atlas levels referring to Karten and Hodos (1967). For abbreviations, see list.

All stained sections were digitized at 20x magnification with a fluorescent microscope system (AxioScan.Z1, Zeiss, Germany). For quantitative analysis, regions of interest were delineated in all sections (about 25 sections per individual) and in both hemispheres and their area was estimated (in mm^2^) with the software ZEN2 (Zeiss, Germany). Following immunoreactive cells were counted manually with the ZEN2 counting tool in all delineated regions: BrdU+, DCX+, BrdU+/NeuN+ and BrdU+/GFAP+. Thus, a value for each region of the number of immunoreactive cells per mm^2^ was determined. Atlas levels for all sections were determined, too. The brain regions and atlas levels for all sections were identified and named in accordance with “The stereotaxic atlas of the brain of the pigeon” (Karten and Hodos, 1967) using the nomenclature recommended by the “Avian Brain Nomenclature Forum” (Reiner et al., 2004). The following telencephalic brain regions were analysed (Figure 1): apical part of the hyperpallium (HA), intercalated part of the hyperpallium (HI), densocellular part of the hyperpallium (HD), mesopallium (M), nidopallium [N, with its subdivisions frontal nidopallium (NF), intermediate nidopallium (NI) and caudal nidopallium (NC)], and hippocampal formation (HF, values of the hippocampal formation were taken from Herold et al., 2019). Firstly, the number of immunoreactive cells/area (mm^2^) was averaged for all analysed structures and secondly according to different atlas levels. For the anterior–posterior analysis of the hyperpallia parts, the number of immunoreactive cells was averaged for the following coordinates ±500 μm (Figure 1 shows the atlas levels of some coronal sections to illustrate the progression from anterior to posterior): A 14.00, A 12.75, A 11.50, A 10.25, A 9.00 and A 7.75 (not for HI and HD). For the mesopallium, data of the coordinates (±500 μm) A 14.25, A 12.75, A 11.25, A 9.75, A 8.25 and A 6.75, and for the nidopallium data of the coordinates (±500 μm) A 13.50, A 10.75, A 8.75, A 6.75 and A 4.50 were used. To determine differences in immunoreactive cells across subdivisions or among subdivisions at different anterior–posterior levels, we first applied a Friedman Repeated Measures Analysis of Variance (FRM ANOVA) for each tested neurogenic marker or marker pair (BrdU+, DCX+, BrdU+/NeuN+ and BrdU+/GFAP+). With this nonparametric test for repeated measures, we could prove whether our groups differ in general, whereby groups mean either the different subdivisions in a first analysis or the different atlas levels in a second analysis. In case of significance, pair-wise comparisons were done with the Wilcoxon Signed Rank test. It allows comparing two samples whose data are not normally distributed. Each subdivision was compared successively to all other regions and each atlas level to all other atlas levels. The level of significance was 5%. The software package SigmaPlot/SigmaStat version 12.0 (Systat Software Inc., United States) was used for all statistic calculations.

To get an impression of the proportion of newborn neurons in the total number of neurons, we have taken the total number of hyperpallium, mesopallium, nidopallium and hippocampal formation from Ströckens et al. (2022) and adjusted them to cells/mm^2^. For this, we used our total area values and calculated the percentage relationship between the total neuron number and the number of newborn neurons (BrdU+/NeuN+ double labelled cells).

## Results

### Qualitative Analysis of Different Stages of Adult Neuro- and Gliogenesis in the Pigeon Telencephalon

**Figure 2:**
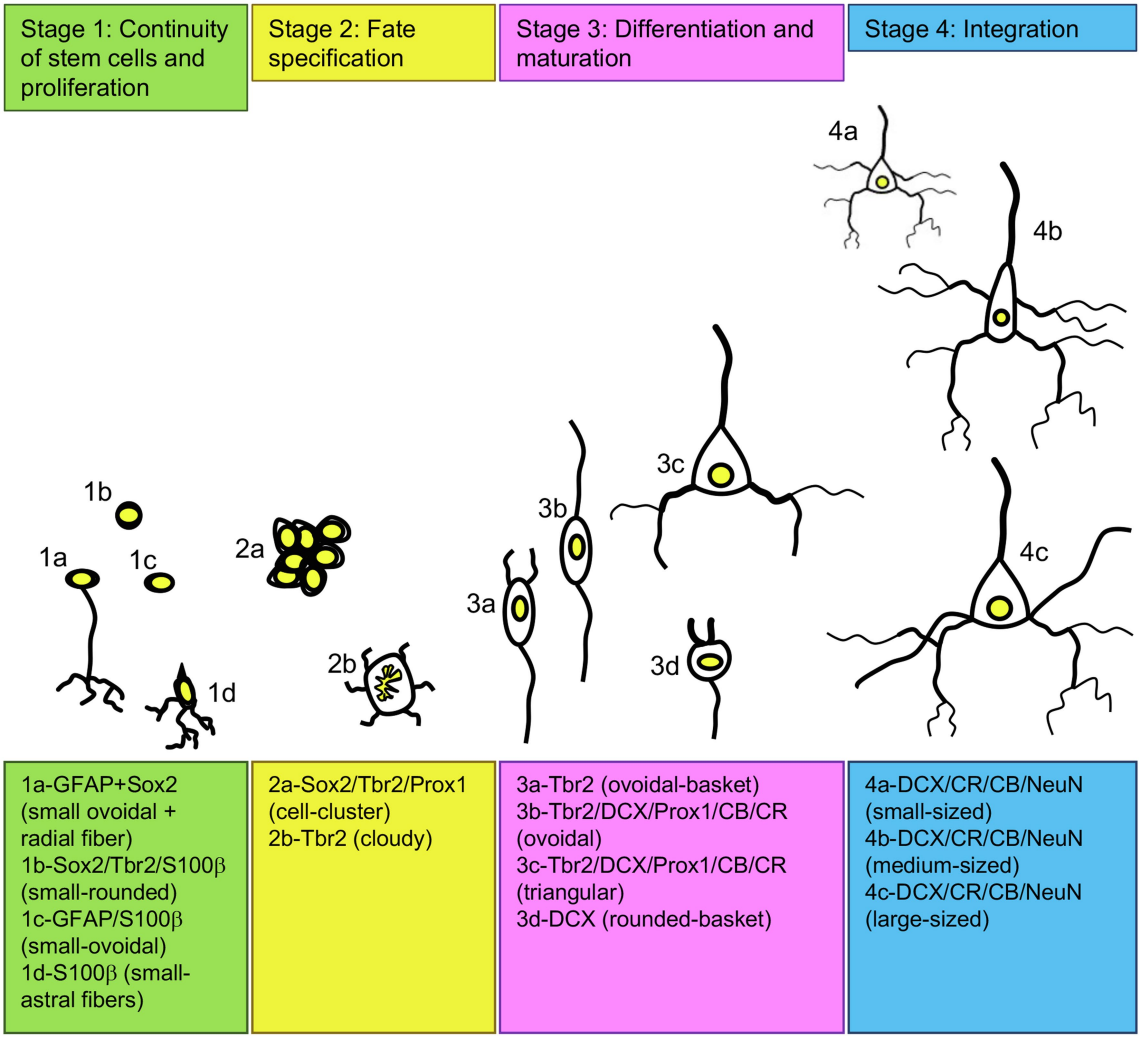
Adult neurogenesis in the pigeon forebrain. Schematic of different developing cell types in the adult pigeon telencephalon that are organized in four stages of neurogenesis based on their shape, size and expression of transcription factors and neurogenic or gliogenic marker proteins. For abbreviations see list.

**Figure 3:**
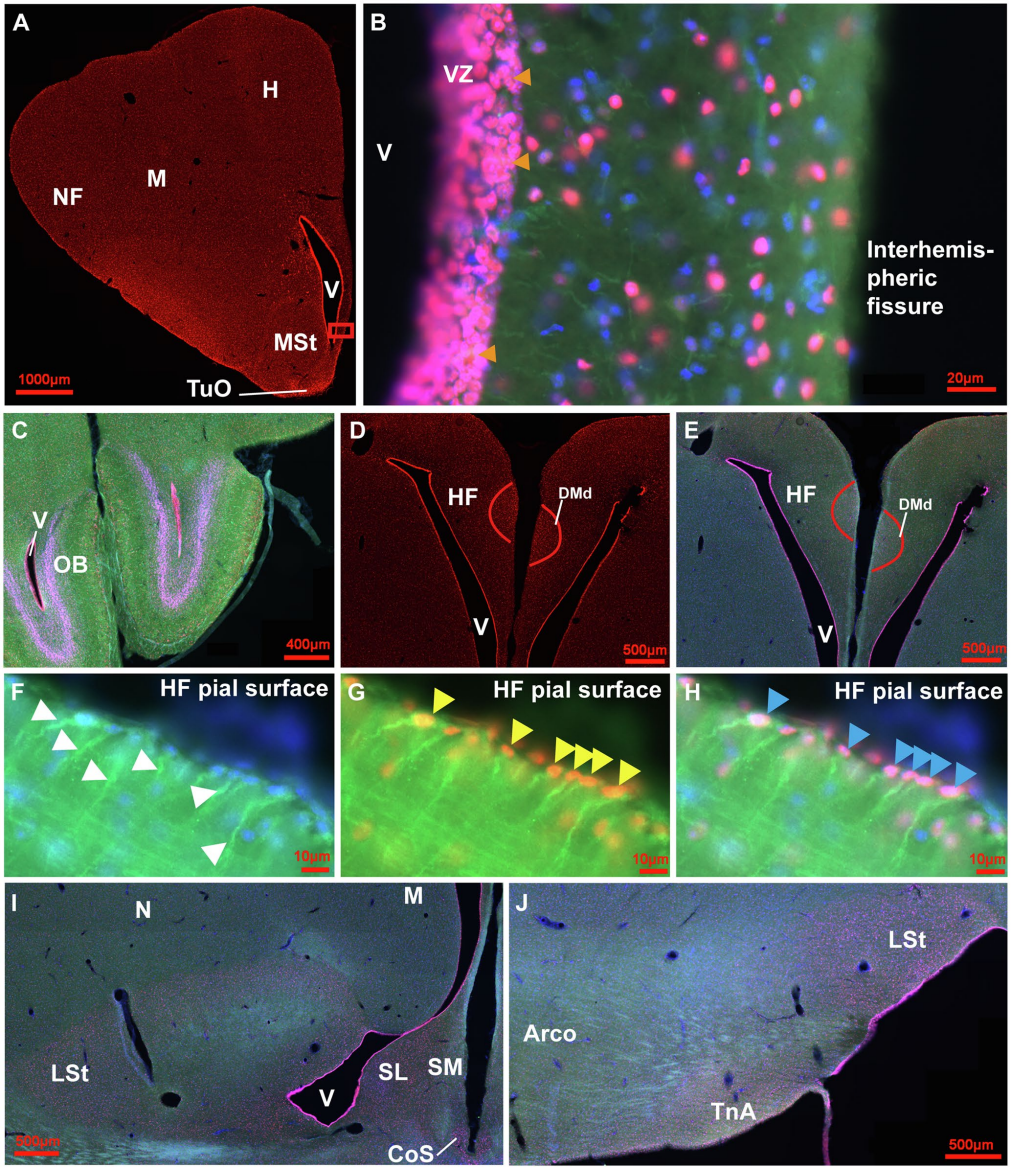
Distribution of Sox2+ cells in the adult pigeon forebrain. **(A)** Sox2 expression (red) in a frontal section at atlas level A13.00 (Karten and Hodos, 1967). Intense labelling can be observed in the ventricular zones, at the pial surface and in TuO. **(B)** Magnification of the ventricular zone (box in A) with triple-staining of Sox2 (red), GFAP (green) and Hoechst (blue). Pinkish cells show double labelling for Sox2 and Hoechst indicating nuclear expression in cells of stage 1 and 2. Yellow/Orange cells show triple labelling for Sox2, GFAP and Hoechst. In addition, green GFAP-positive radial fibres from the ventricular zone can be seen. Orange arrowheads point to cell clusters of stage 2. **(C)** Triple staining in the OB at atlas level A14.50 showing intense labelling in the ventricular zone and in the internal granular layer. **(D)** Sox2 expression in the HF with intense labelling of cells in DMd and the ventricular zone at atlas level A7.75. **(E)** Triple-labelling in the HF shown in **(D)**. **(F-H)** Magnifications of the pial surface of the HF at atlas level A7.00. GFAP+ (green)/Hoechst+(blue) radial cells with radial fibres (white arrowheads) shown in F. GFAP+/Sox2+ (yellow) radial cells (yellow arrowheads) shown in **G**. GFAP+/Sox2+/Hoechst+ (pink/orange) radial cells (blue arrowheads) shown in **H**. **(I,J)** Triple-staining of Sox2 (red), GFAP (green) and Hoechst (blue) of frontal sections at atlas level A7.75 **(I)** and A6.25 **(J)** that show enhanced positive signals in the LSt, the ventricular zone, SL, SM, CoS and TnA in comparison to less signals in N, M and Arco. For abbreviations see list.

The qualitative analysis of neurogenic and gliogenic stage markers resulted in a first comprehensive overview of cell types that express different fate and stage markers. We classified four different stages (Figure 2 1st stage: continuity of stem cells/proliferation, 2nd stage: fate specification, 3rd stage: differentiation/maturation, 4th stage: integration). Generally, GFAP, S100ß, DCX and NeuN co-labelling with BrdU was observed showing that stem cells actively divide and generate new stem cells, new glial cells and new neurons in the adult pigeon forebrain. Thereby, the combined analysis of Sox2 and GFAP demonstrated co-labelling and thus indication of actively dividing stem cells (stage 1). Those were detected at high amounts in the ventricular zones through the whole anterior–posterior axis (Figures 3A–E) and at the pial surface (Figures 3F–H). The stem cells additionally have radial fibres that are GFAP positive (Figures 3F–H). Further, small-rounded Sox2+ cells were distributed all over the forebrain with very high densities in the olfactory tubercle (TuO; Figure 3A), olfactory bulb (OB; internal plexiform and granular cell layer; Figure 3C), in the hippocampal formation (DMd; Figures 3D,E), the septal nuclei (SL + SM, Figure 3I), the lateral striatum (LSt; Figures 3I,J) and the nucleus taenia of the amygdala (TnA; Figure 3J). GFAP and Sox2 co-stained cells were assigned to stage 1 because they further possessed radial fibres and conclusively can be considered as active radial stem cells. Small-ovoidal GFAP+/BrdU+ and S100ß+/BrdU+ cells were found all over the forebrain that resembles further gliogenesis. Thereby, S100ß + positive cells that were observed in the tissue had typically astro-like processes, representing astrocytes. Another type of S100ß + cell with astral fibres and an endfoot process was detected in the OB (external plexiform and mitral cell layer) in close proximity to a rounded S100ß+/BrdU+ cell and a BrdU+ cell representing a “niche”-figure (Supplementary Figures S2A,B).

**Figure 4:**
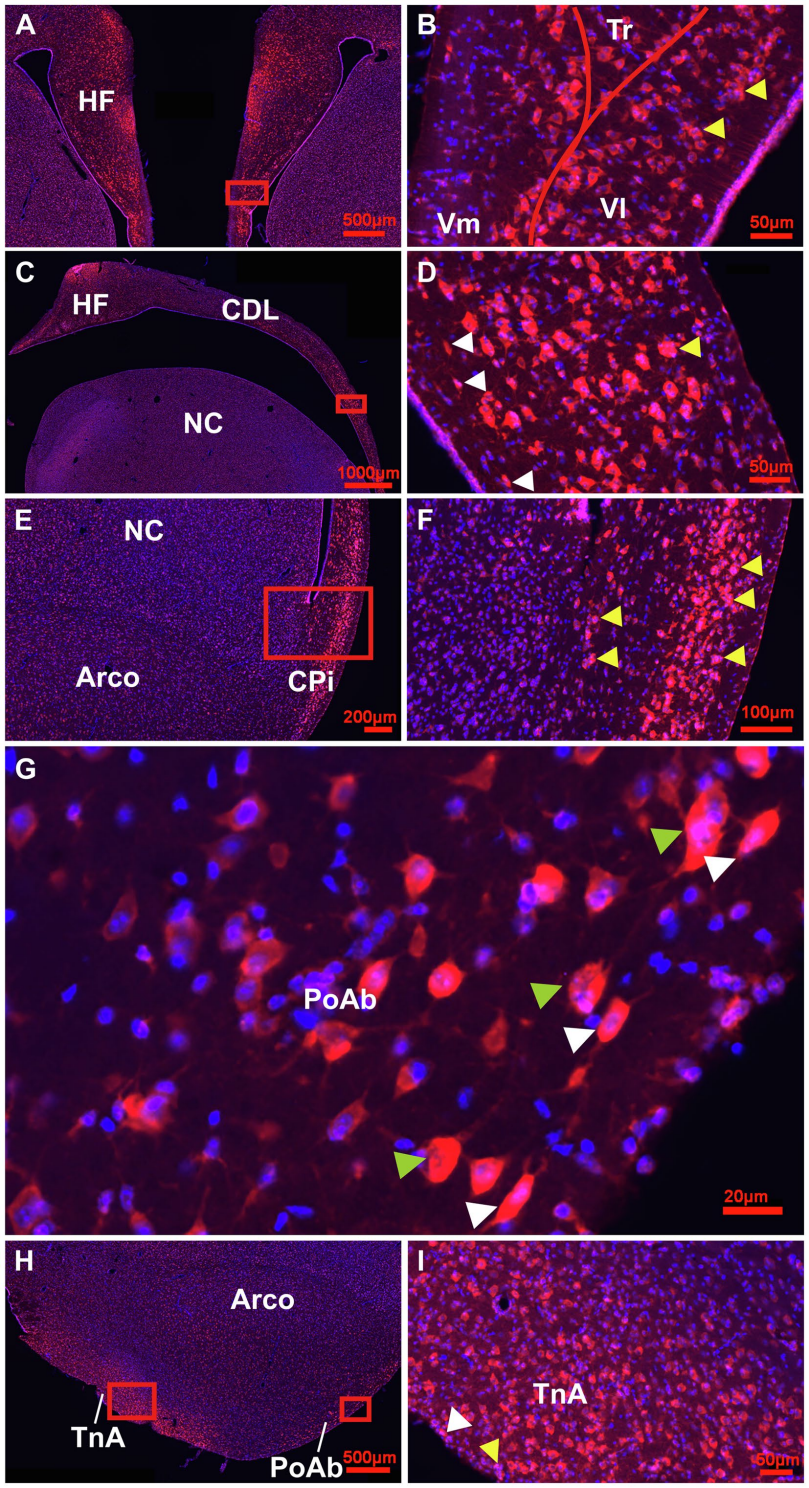
Detailed overview of Tbr2+ cells in the adult pigeon forebrain. **(A)** Detail of the HF at atlas level A8.00 showing intense labelling of Tbr2 + (red)/Hoechst+(blue), i.e., double labelled pinkish cells in the DMd region and V-region of the HF. **(B)** Magnification of the Box in **(A)** showing a different cellular location of Tbr2+ cells in the V-region with high densities in VL (stage 2–3) and the ventricular zone (stage1). Cell clusters of stage 2 are indicated by yellow arrowheads. **(C)** Detail of the HF and CDL at atlas level A.6.00 with pinkish TBr2+/Hoechst+ cells accumulated in DMd and ventral CDL shown enlarged from the box in **(D)**. Here, white arrowheads indicate migrating cells of stage 3 and yellow arrowheads cell clusters of stage 2 **(D)**. **(E)** Detail of Tbr2+ expression in CPi cells, enlarged in **(F)**. Yellow arrowheads indicate cell clusters at both sides, close to ventricular zone and to the granular layer of the Cpi, which is close to pial surface **(F)**. **(G)** High magnification image of Tbr2+/Hoechst+ cells of stage 2 cloudy (mitotic) cells, indicated by green arrowheads and stage 3 migrating (ovoidal) cells, indicated by white arrowheads along the outer band of PoAb (position of box in **H**). **(H)** Detail of Tbr2+ expression at the level of TnA, PoAb and Arco that is enlarged for TnA in **(I)**. **(I)** Tbr2+/Hoechst+ cells in TnA showing stage 2 cluster cells (yellow arrowhead) and stage 3 ovoidal, migrating cells (white arrowhead). Generally, Tbr2 is found in the cell somata, but is also present in the nuclei, particularly in the small cells of the ventricular zones (double-labelled cells Tbr2+/Hoechst+ appear pinkish).

**Figure 5:**
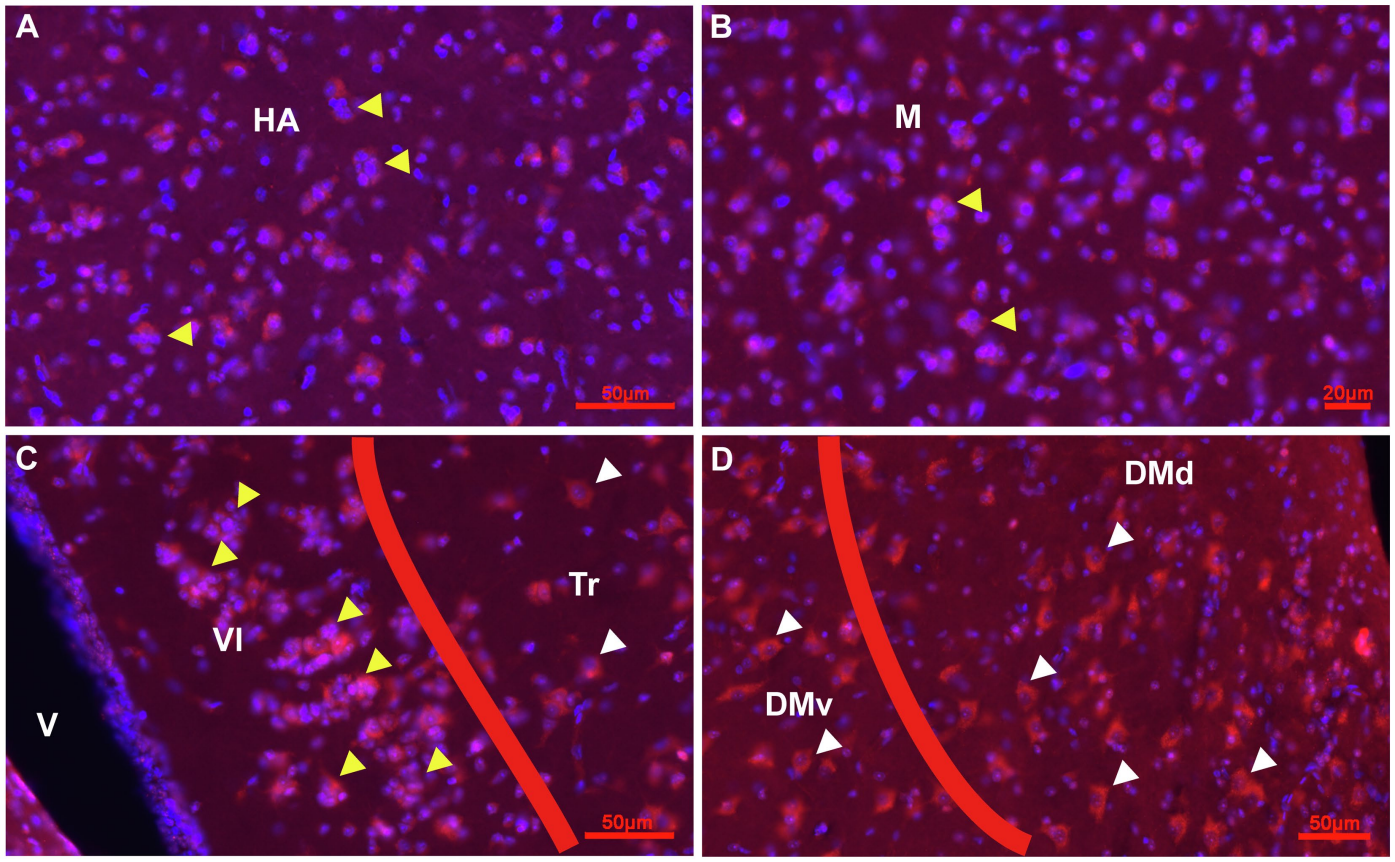
Prox1 expression in combination with Hoechst staining in the adult pigeon forebrain. **(A)** Detail of Cluster Prox1 + (red)/Hoechst+ (blue), i.e., red-pinkish cells of stage 2 in the parenchym of HA (yellow arrowheads) at atlas level 12.25. **(B)** Detail of Cluster Prox1+ cells of stage 2 in the parenchym of M (yellow arrowheads) at atlas level A7.75. **(C,D)** Prox1+ cells in the HF at atlas level A7.75. Clusters occur mainly in VL (yellow arrowheads), while Tr, DMv and DMd exhibited Prox1+ cells of stage 3 and 4 (white arrowheads). Apart from clusters, Prox1 immunostaining was mainly observed in the soma of cells.

**Figure 6:**
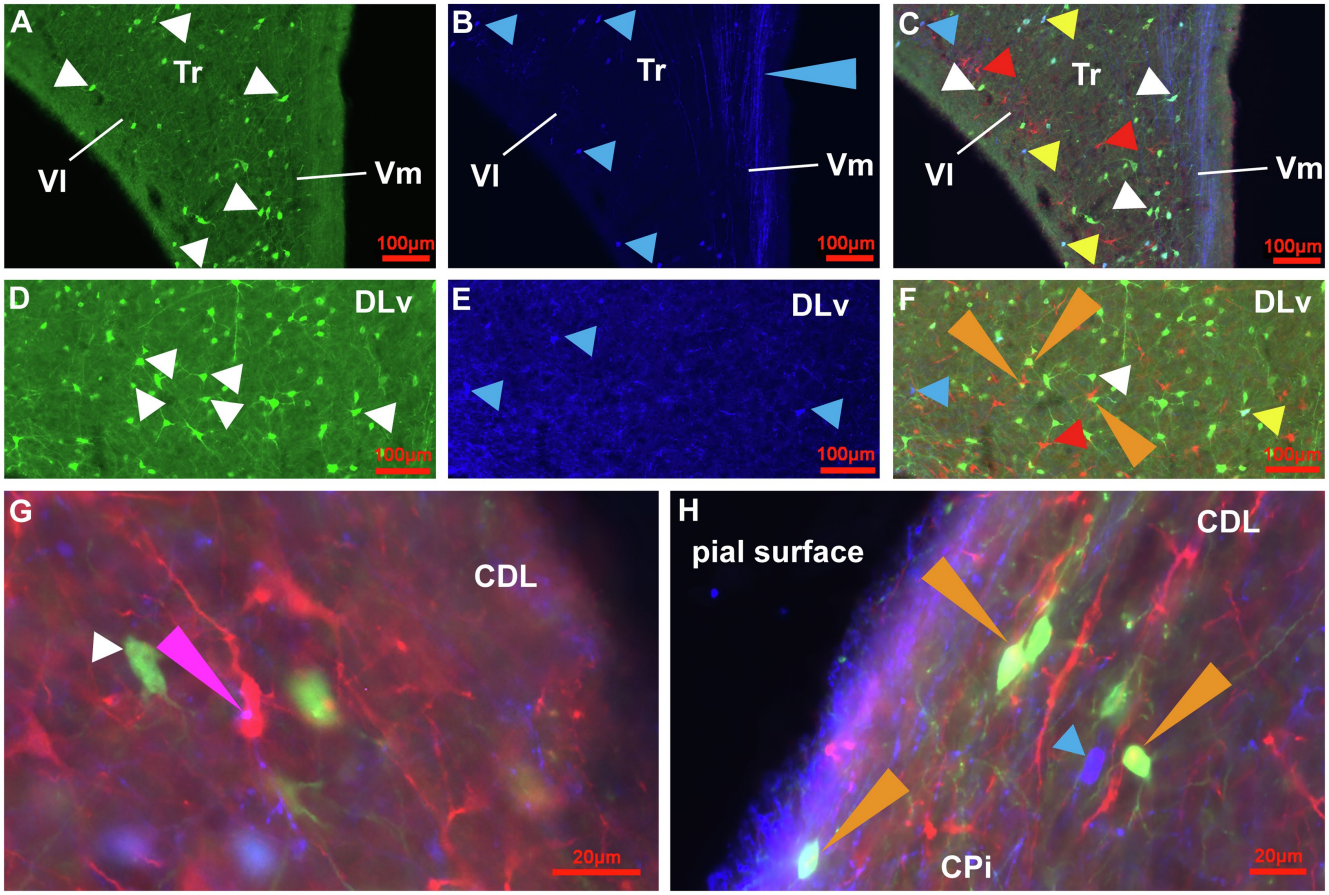
Immunoreactive cell types for CB, CR and DCX in the HF, CDL and CPi of the adult pigeon. **(A-C)** Detail of the V-Region of the HF at atlas level A4.75. CB+ (green) cells with different size, shape and fiber architecture (white arrowheads) are shown in **(A)**. CR+ (dark blue) cells (blue arrowheads) and CR+ fibers (blue long arrowhead) are shown in **(B)**. **(C)** Overlay of triple-staining for CB, CR and DCX (red). White arrowheads point to CB+ cells (green), blue arrowheads point to CR+ cells (dark blue). Red arrowheads point to DCX+ cells (red). Yellow arrowheads point to CB+/CR+ (light blue-green) double labelled cells. **(D-F)** Detail of DLv of the HF at atlas level A7.00. CB+ (green) cells with different size, shape and fiber architecture (white arrowheads) are shown in **(D)**. CR+ (dark blue) cells (blue arrowheads) are shown in **(E)**. **(F)** Overlay of triple-staining for CB, CR and DCX (red). White arrowhead points to CB+ cells (green), blue arrowhead points to CR+ cells (dark blue). Red arrowhead points to DCX+ cells (red). Yellow arrowhead points to CB+/CR+ (light blue-green) double labelled cells. Orange long arrowheads point to CB+/DCX+ (yellow) double labelling. **(G)** Magnification of cell types and fibers in CDL at atlas level A7.00. The white arrowhead points to a CB+ (green) cell of stage 3, the pink long arrowhead points to CR+(blue)/DCX+(red) double labelling showing that CR + fibers contact DCX+ ovoidal cells of stage 3 appearing in pink. **(H)** Magnification of cell types and fibers in the transition area between CPi and CDL at atlas level A6.50. The blue arrowhead points to a CR+ (blue) ovoidal cell of stage 3. The orange arrowheads point to CB+(green)/DCX+(red) double labelled cells appearing in yellow.

At the next stage, Tbr2 was used to detect non-radial progenitor cells. Tbr2+ cells had different sizes and shapes. High densities were observed in the HF (Figures 4A–C), in the area corticoidea lateralis (CDL, Figures 4C,D), in the cortex piriformis (CPi, Figures 4E,F), the posterior amygdala, pars basalis (PoAb, Figures 4G,H) and the TnA (Figures 4H,I). At stage 1 they overlap in size and shape with Sox2+ cells. At stage 2, Tbr2+ and Sox2+ cells were found in cell clusters in different regions close to the ventricular zone, concentrated in the HF and CPi and randomly allocated in different forebrain structures (Figures 3B, 4B,D,F,I). Beside the mentioned areas, these cell clusters have been also detected in the apical hyperpallium and in the mesopallium expressing the neuronal lineage marker Prox1 (Figures 5A–C). Another Tbr2+ cell type has been detected which exhibits typical figures of mitosis (Figure 4G). Those “cloudy” cells were often observed in close proximity to stage 3 ovoidal cells (Figures 4G, 6). Here, two types of ovoidal (i.e., migrating cells) have been detected. The first type, the ovoidal-basket cell, expressed Tbr2 and has yet not been found to express one of the other tested neurogenic markers (Figures 4G, 6). It can be distinguished from a second type, the typical ovoidal cell that has been found to express Tbr2, DCX, Prox1, CB and CR (Figures 3, 4, 6). Thereby, regularly, ovoidal cells were observed to be DCX+. Beside the expression of several neuronal lineage markers, the two types also differ within their processes. The ovoidal-basket cells exhibit one stronger process and two smaller processes that look like handles, while the typical ovoidal cells usually exhibit two stronger processes facing each other. Both types further differ in their cell body size from the small-ovoidal cells that have been detected at stage 1, which were typically about 5 μm, while ovoidal cells at stage 3 were about 10–15 μm in their length. Tbr2+ cells were associated to stage 1, 2 and 3 of neurogenesis because similarly to Sox2+ small-rounded cells, Tbr2+ cells were found concentrated in the ventricular zones and regionally distributed in the forebrain (stage 1). Tbr2+ cells of stage 2 were detected in cell clusters that could be also observed with Sox2 or Prox1 immunostaining. Those clusters were either concentrated close to the ventricular zones, to superficial sites of the forebrain or neurogenic regions like the HF or the CPi but also occurred randomly in the parenchyma, for example, in the apical hyperpallium or the mesopallium.

**Figure 7:**
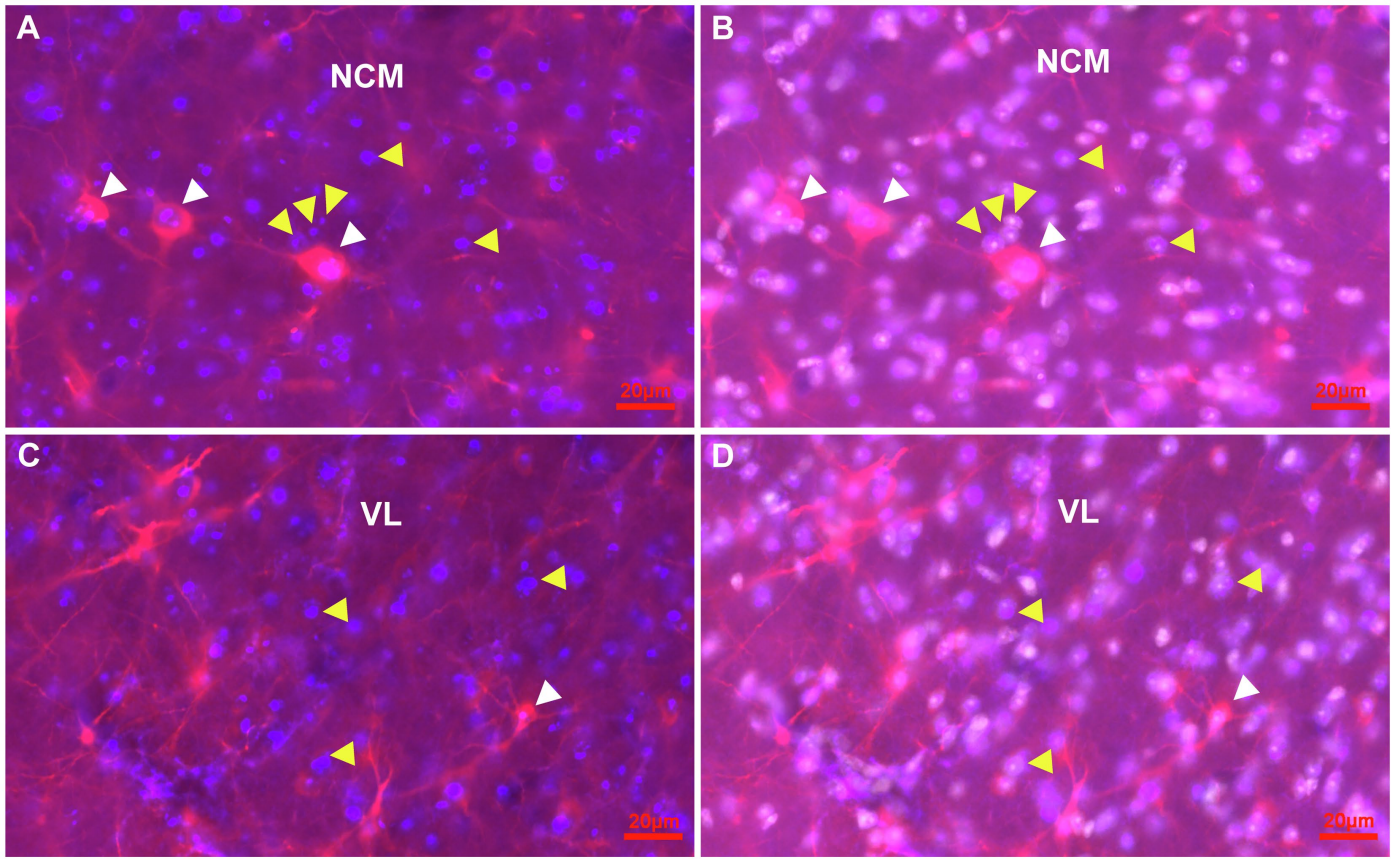
Representative pictures of the parenchym of the NCM and the VL of the HF with cells immunostained for Ki67 and DCX in combination with Hoechst at atlas level A4.50. **(A,B)** Detail of Ki67 + (blue) cells (yellow arrowheads) and DCX+(red) cells (white arrowheads) in the NCM. A stage 3 ovoidal DCX+/Ki67+ double labelled cell (pink) is shown (most left), while the other two larger DCX+/Ki67+ double labelled cells show pronounced fibres akin like multipolar stage 4 cells **(A)**. Hoechst staining (white) revealed that Ki67 is located in the nuclei of cells **(B)**. **(C,D)** Detail of Ki67 + (blue) cells (yellow arrowheads) and DCX+(red) cells (white arrowheads) in the VL region of the HF. A typical multipolar DCX+/Ki67+ double labelled cell (pink) of stage 4 of the VL region (white arrowhead). Hoechst staining (white) showed that Ki67+ is expressed in the nuclei **(D)**.

**Figure 8:**
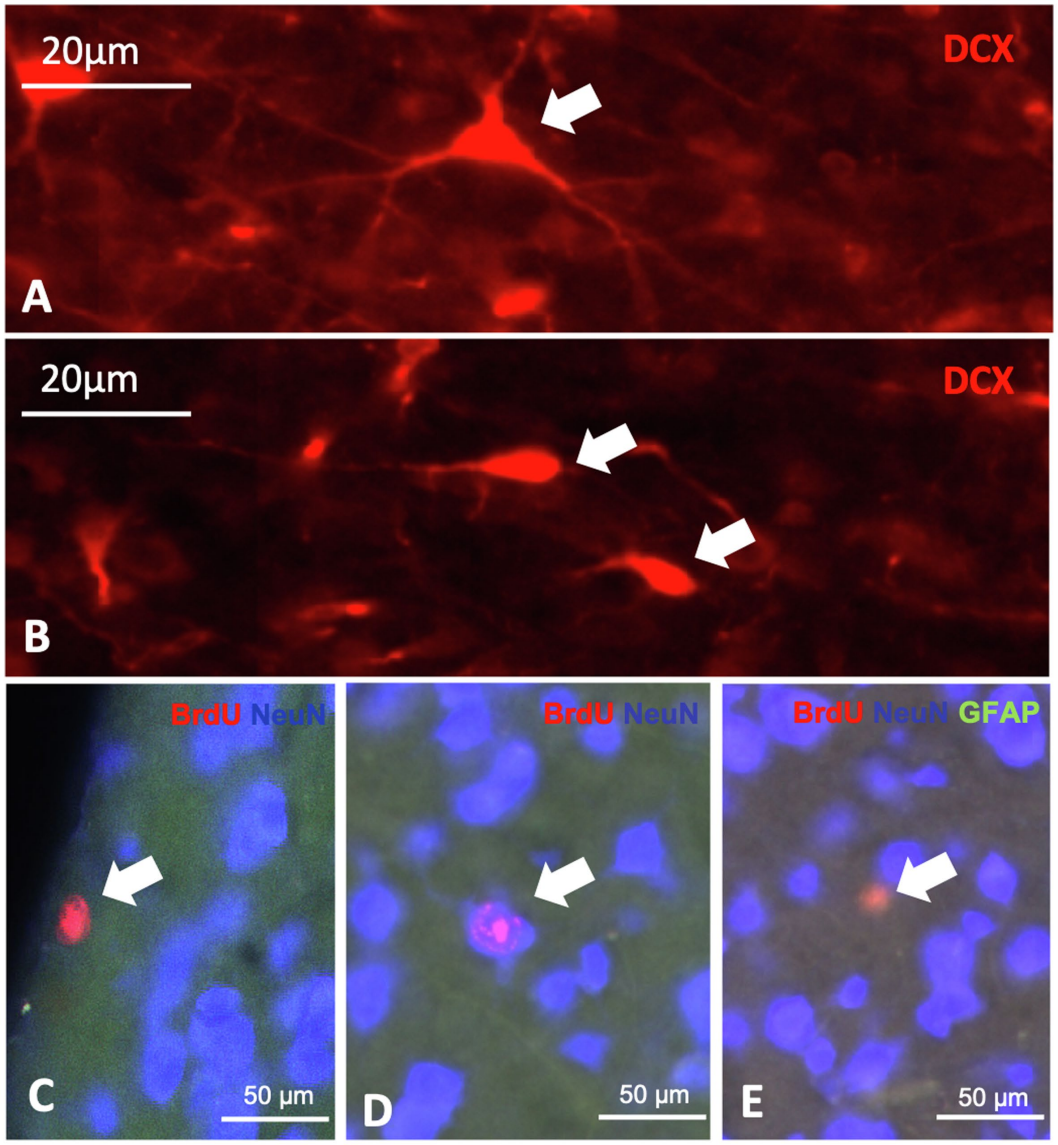
Expression of neurogenic markers in the telencephalon of the pigeon. **A + B**: different DCX positive cells, characterized by shape, the triangular cell **(A)** and the ovoid cell **(B)**. **c-e**: BrdU, NeuN and GFAP triple labelling showing BrdU positive cells **(C)**, double labelled BrdU/NeuN cells **(D)** and double labelled BrdU/GFAP cells **(E)**.

The other prominent cell type at stage 3 was the triangular type, which has been also found to be Tbr2+, DCX+, Prox1+, CB+ and CR+. The triangular type of stage 3 differed mostly with respect to its branches, which were less prominent when compared to the different stage 4 multipolar, matured cell types (Figures 4B,D,F,G,I, 5C,D, 6A–F, 7, 8A). Further, DCX+/CB+ and DCX+/CR+ double labelled cells were observed (Figures 6A–H). Thereby, also fibre contacts were found with different co-labelling of DCX+/CB+ and DCX+/CR+ for example in the CDL (Figures 6G,H). Triangular and ovoidal DCX+ cells were quantified as migrating and proliferating/differentiating neurons in the different telencephalic areas as described below (Figures 2, 8A,B; Table 1; Supplementary Table S5). In the nidopallium, a third type of DCX+ cell was observed that has yet not been detected in the other analysed areas: the rounded-basket type (Supplementary Figure S3). It showed two strong, divergent processes at the same side of the cell body. Contrary to the typical ovoidal DCX+ cell, the cell body of these basket DCX+ cells appeared rounded and possessed a larger diameter.

**Table 1:** Distribution of BrdU+, BrdU+/NeuN+ and BrdU+/GFAP+ cells/mm^2^ in different telencephalic regions of the pigeon.

	BrdU all	BrdU+	BrdU+/NeuN+	BrdU+/GFAP+
Apical Hyperpallium	11.28 ± 1.98	4.22 ± 1.48	1.79 ± 0.46	5.28 ± 0.88
Intercalated Hyperpallium	14.36 ± 2.07	4.71 ± 1.31	2.62 ± 0.68	7.03 ± 1.35
Densocellular Hyperpallium	11.56 ± 1.36	3.56 ± 0.70	2.10 ± 0.53	5.91 ± 1.01
Mesopallium	11.29 ± 1.60	3.89 ± 0.95	2.00 ± 0.50	5.40 ± 1.10
Frontal Nidopallium	8.98 ± 1.36	1.69 ± 0.39	0.89 ± 0.23	6.40 ± 1.08
Intermediate Nidopallium	5.71 ± 0.71	1.08 ± 0.21	0.77 ± 0.15	3.85 ± 0.63
Caudal Nidopallium	3.87 ± 0.47	1.33 ± 0.21	1.24 ± 0.26	1.29 ± 0.23
Hippocampal formation	17.27 ± 2.35	3.72 ± 1.13	1.75 ± 0.39	11.80 ± 2.42

‘BrdU all’ means the sum of all BrdU labelled cells (single and double labelled). Values are means±S.EM.

**Figure 9:**
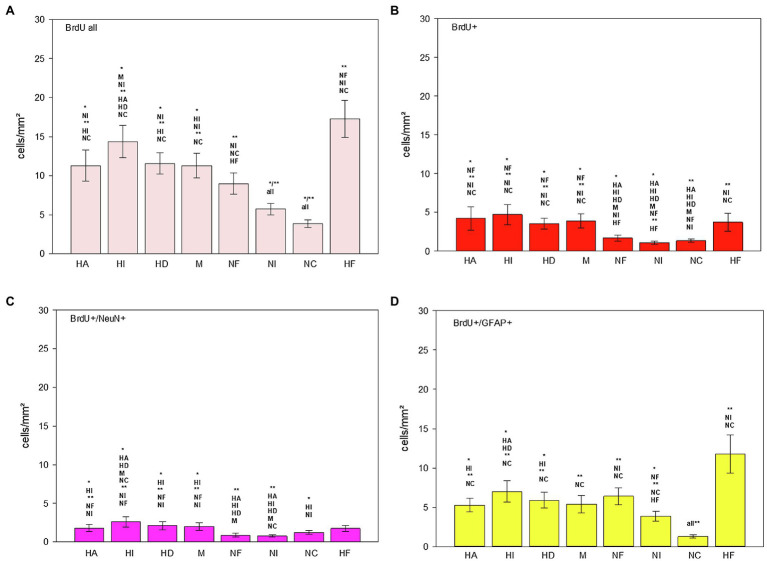
Distribution of BrdU+ **(B)**, BrdU+/NeuN+ **(C)** and BrdU+/GFAP+ **(D)** cells/mm^2^ in different regions of the telencephalon of the pigeon (*n* = 9). “BrdU all” **(A)** means the sum of all BrdU labelled cells (single and double labelled). Bars represent mean ± SE. Asterisks indicate significant differences (Wilcoxon Signed Rank Test, ^*^p < 0.05, ^**^p < 0.01).

**Figure 10:**
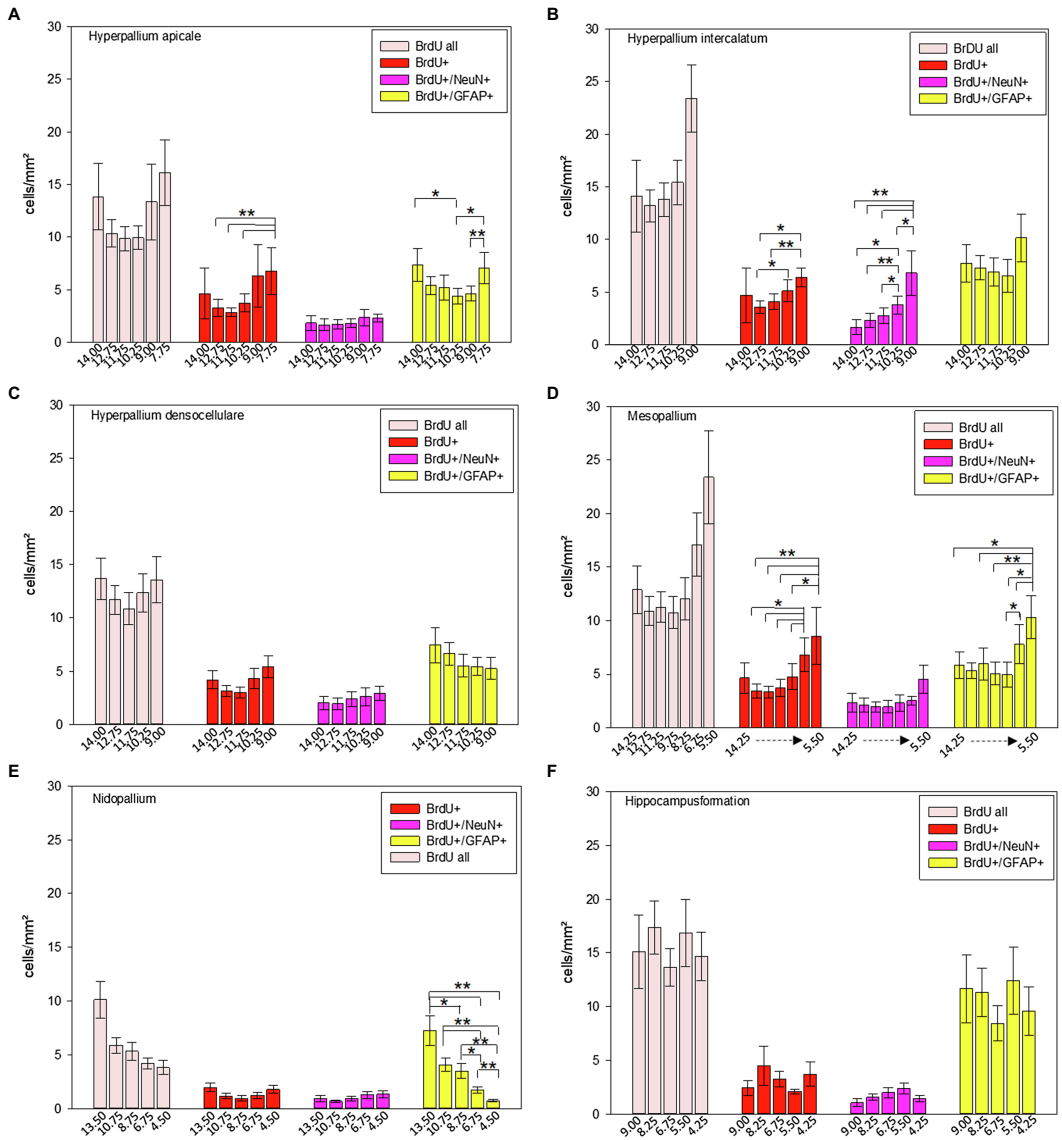
Distribution of BrdU+, BrdU+/NeuN+ and BrdU+/GFAP+ cells/mm^2^ of different atlas levels along the anterior–posterior axis in different regions of the pigeon (*n* = 9). **(A)** Hyperpallium apicale, **(B)** Hyperpallium intercalatum, **(C)** Hyperpallium densocellulare, **(D)** Mesopallium, **(E)** Nidopallium, **(F)** Hippocampal formation. “BrdU all” means the sum of all BrdU labelled cells (single and double labelled). Atlas level A13.50 in nidopallium represents NF, A10.75 and A8.75 NI and A6.75 and A4.50 NC. Bars represent mean ± SE. Asterisks indicate significant differences (Wilcoxon Signed Rank Test, ^*^p < 0.05, ^**^p < 0.01).

Cells at stage 4 were all multipolar and differed in their size, shape, number of processes and length of their processes (Figures 6, 7). They have been found to express CB and CR as described earlier, for example, in the hyperpallium (Heyers et al., 2008), in the striatum (Bruce et al., 2016) and shown here in the hippocampal formation (Figures 6A–F). A small number of cells at stage 4 were further CB+/CR+ double labelled (Figures 6A–F). Stage 4 cell types also expressed NeuN, and were quantified as adult newborn neurons in combination with BrdU double-labelling in the different brain areas as described below (Figures 8C, 9, 10; Table 1; Supplementary Table S2).

To address the question of proliferating and maturing cells in general, a co-staining of Ki67 and DCX was conducted (Figures 7A–D). Here it was observed that Ki67+ cells were homogenously distributed throughout the entire forebrain and that labelling was limited to the cell nucleus. Ki67+/DCX+ co-expression was further observed in different brain areas like in the nidopallium and in the hippocampal formation (Figures 7A–D).

### Quantitative Analysis of Adult Neuro- and Gliogenesis in the Pigeon Telencephalon

BrdU labelled cells were found in all analysed brain structures, as well as double labelled BrdU/NeuN and double labelled BrdU/GFAP cells (Table 1; Figures 8C,E, 9). There are significant regional differences in their spatial distribution [FRM ANOVA, BrdU+: *χ*^2^(*n* = 9, df = 7) = 46.89, *p* < 0.001; BrdU+/NeuN+: *χ*^2^(*n* = 9, df = 7) = 32.78, *p* < 0.001; BrdU+/GFAP+: *χ*^2^(*n* = 9, df = 6) = 34.94, *p* < 0.001].

The highest numbers of BrdU labelled cells and newborn glial cells (BrdU+/GFAP+) were detected in the hippocampal formation (Table 1; Figures 9A,D; Wilcoxon Signed Rank Test, HF, BrdU all: NF, NI, NC, *p* < 0.01; BrdU+/GFAP+: NI, NC, *p* < 0.01), followed by the intercalated hyperpallium (Wilcoxon Signed Rank Test, HI, BrdU all: M, NI, *p* < 0.05, HA, HD, NC *p* < 0.01; BrdU+/GFAP+: HA, HD, *p* < 0.05, NC, *p* < 0.01). The intercalated hyperpallium also showed the highest numbers of newborn neurons (Figure 9C; Wilcoxon Signed Rank Test, HI, BrdU+/NeuN+, HA, HD, M, NC, *p* < 0.05, NF, NI, *p* < 0.01). The nidopallium generally showed low numbers of BrdU labelled cells, with the lowest numbers in the caudal nidopallium (Figure 9A; Wilcoxon Signed Rank Test, NC, BrdU all: NF, *p* < 0.05, all other structures, *p* < 0.01) and the lowest number of BrdU+/NeuN+ cells in the intermediate nidopallium (Wilcoxon Signed Rank Test, NI, BrdU+/NeuN+: NC, *p* < 0.05, HA, HI, HD, M, NC, *p* < 0.01). Detailed statistical values can be found in the Supplementary Table S1.

Generally, the total number of newborn glial cells exceeded the total number of newborn neurons (Wilcoxon Signed Rank Test, *T* = 36.00, *p* = 0.008) and the total number of BrdU+ cells (newborn cells which were neither neurons nor glial cells) exceeded the total number of newborn neurons or glial cells (Wilcoxon Signed Rank Test, *T* = 36.00, *p* = 0.008).

Along the anterior–posterior axis, BrdU labelled cells showed a different distribution in the apical hyperpallium [FRM ANOVA, *χ*^2^(*n* = 9, df = 5) = 15.03, *p* = 0.010, Figure 10A], the intercalated hyperpallium [FRM ANOVA, *χ*^2^(*n* = 9, df = 4) = 9.60, *p* = 0.048, Figure 10B] and the mesopallium [FRM ANOVA, *χ*^2^(*n* = 9, df = 5) = 12.37, *p* = 0.030]. BrdU+/NeuN+ cells just showed anterior–posterior variations in the intercalated hyperpallium [FRM ANOVA, *χ*^2^(*n* = 9, df = 4) = 20.53, *p* < 0.001]. For BrdU+/GFAP+ cells, we found anterior–posterior variations in the apical hyperpallium [FRM ANOVA, *χ*^2^(*n* = 9, df = 5) = 12.11, *p* = 0.033, Figure 10A], the mesopallium [FRM ANOVA, *χ*^2^(*n* = 9, df = 6) = 17.24, *p* = 0.008, Figure 10D] and the nidopallium in total [FRM ANOVA, *χ*^2^(*n* = 9, df = 4) = 26.40, *p* < 0.001, Figure 10E].

In HA, HI and M, BrdU+ cells dropped at the most posterior part around atlas level A7.75 (HA), A9.00 (HI), A6.75 and A5.50 (M; Figures 10A,B,D; Supplementary Tables S2 and S3). The distribution of newborn neurons (BrdU+/NeuN+) in the intercalated hyperpallium showed a clear increase from anterior to posterior with highest numbers at atlas levels A10.75 and A9.00 (Figure 10B; Supplementary Tables S2 and S3). In the distribution of newborn glial cells (BrdU+/GFAP+) there were no clear trends (increase or decrease) along the anterior–posterior axis except for the nidopallium. Here, newborn glial cells decreased from anterior to posterior with the highest numbers at level A13.50 (mostly NF) and the lowest numbers at atlas level A4.25, where NC is located (Figure 10E; Supplementary Tables S2 and S3). Remaining individual values and statistical values can be found in the Supplementary Tables S2 and S3.

Generally, the proportion of newborn neurons of the total number of neurons is small (Table 2) and less than 1% for all structures. Here, the hyperpallium showed the highest percentage with 0.088% and the nidopallium the lowest with 0.024% of total number of neurons.

**Table 2:** Total number of neurons (data from Ströckens et al., 2022, adjusted to mm^2^) and number of newborn neurons (BrdU+/NeuN+ double labelled cells/mm^2^) including their relationship in different telencephalic regions of the pigeon.

	Total neurons	Newborn neurons	%
Hyperpallium	2451.20	2.17 ± 0.56	0.088
Mesopallium	3335.77	2.00 ± 0.50	0.060
Nidopallium	4097.01	0.97 ± 0.21	0.024
Hippocampal formation	4461.41	1.75 ± 0.39	0.039

Hyperpallium includes HA, HI and HD, nidopallium includes NF, NI and NC. Values of newborn neurons are mean±S.E.

### Quantitative Analysis of Differentiation and Proliferation of Neurons in the Adult Pigeon Telencephalon

DCX labelled cells were found in all analysed brain structures and can be distinguished in triangular-shaped and ovoidal-shaped cells (Table 3; Figures 8A,B). There are significant regional differences in their spatial distribution [FRM ANOVA, DCX+: *χ*^2^(*n* = 9, df = 7) = 49.22, *p* < 0.001; DCX-tri: *χ*^2^(*n* = 9, df = 7) = 40.70, *p* < 0.001; DCX-ovo: *χ*^2^(*n* = 9, df = 6) = 54.41, *p* < 0.001].

**Table 3:** Distribution of DCX+ cells/mm^2^ in different telencephalic regions of the pigeon.

	DCX all	DCX tri	DCX ovo
Apical Hyperpallium	23.80 ± 2.30	16.71 ± 2.21	7.14 ± 0.51
Intercalated Hyperpallium	26.78 ± 4.22	19.67 ± 3.81	7.14 ± 0.54
Densocellular Hyperpallium	15.68 ± 1.81	11.58 ± 1.56	4.15 ± 0.39
Mesopallium	16.01 ± 1.86	12.26 ± 1.68	4.08 ± 0.27
Frontal Nidopallium	104.32 ± 17.68	49.02 ± 7.83	54.65 ± 9.90
Intermediate Nidopallium	108.05 ± 14.65	52.03 ± 6.01	55.34 ± 8.69
Caudal Nidopallium	81.82 ± 8.60	40.55 ± 2.72	40.98 ± 6.30
Hippocampal formation	37.99 ± 3.62	15.61 ± 0.61	22.38 ± 3.61

Two different types of DCX+ cells were recognized, triangular (tri) and ovoid (ovo) cells. Values are means±S.E.

**Figure 11:**
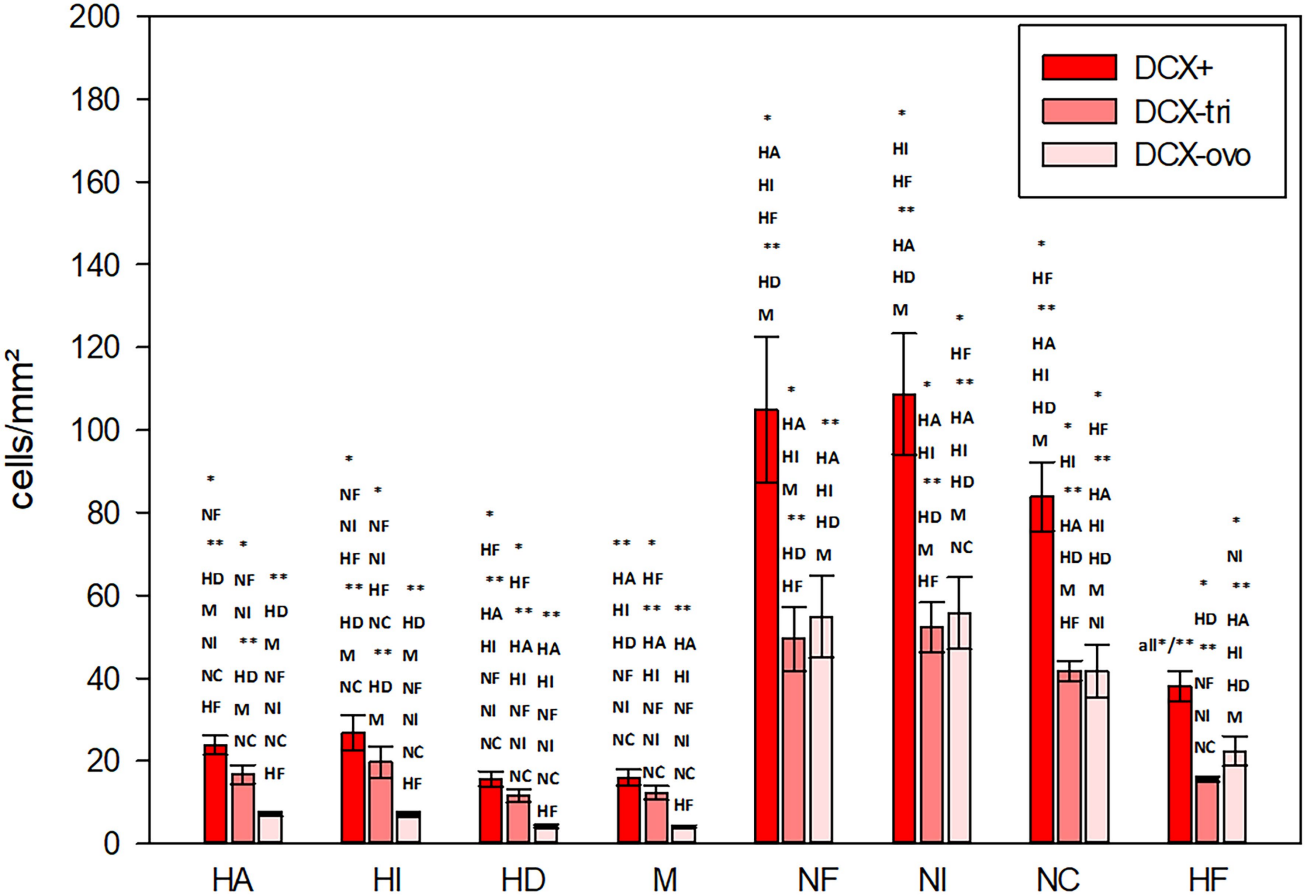
Distribution of DCX+ cells/mm^2^ in different regions of the telencephalon of the pigeon. Two different types of DCX+ cells were recognized, triangular (−tri) and ovoid (−ovo) cells. Bars represent mean ± SE. Asterisks indicate significant differences (Wilcoxon Signed Rank Test, ^*^p < 0.05, ^**^p < 0.01).

The highest numbers of DCX labelled cells (DCX+) were detected in the nidopallium, and here in the intermediate nidopallium (Table 3, Figure 11; Wilcoxon Signed Rank Test, HI, HF, *p* < 0.05, HA, HD, M, *p* < 0.01). The other two nidopallial areas NF and NC also showed a significant higher number of DCX positive cells compared to hyperpallial areas, mesopallium and hippocampal formation (Wilcoxon Signed Rank Test, NF: HA, HI, HF, *p* < 0.05, HD, M, *p* < 0.01; NC: HF, *p* < 0.05, HA, HI, HD, M, *p* < 0.01). The lowest number of DCX positive cells was found in the densocellular hyperpallium (Figure 11; Wilcoxon Signed Rank Test, HF, *p* < 0.05, HA, HI, NF, NI, NC, *p* < 0.01). In the hyper- and mesopallium the number of triangular cells exceeded the number of ovoidal cells (Wilcoxon Signed Rank Test, HA, HI, HD, M: *T* = −45.00. *p* = 0.004), in nidopallium and hippocampal formation the ratio is balanced (Wilcoxon Signed Rank Test, HF, NI: *T* = 25.00, *p* < 0.164, NC: *T* = 11.00, *p* < 0.57) or vice versa (Wilcoxon Signed Rank Test, NF: *T* = 35.00, *p* < 0.039). Detailed statistical values can be found in the Supplementary Table S4.

**Figure 12:**
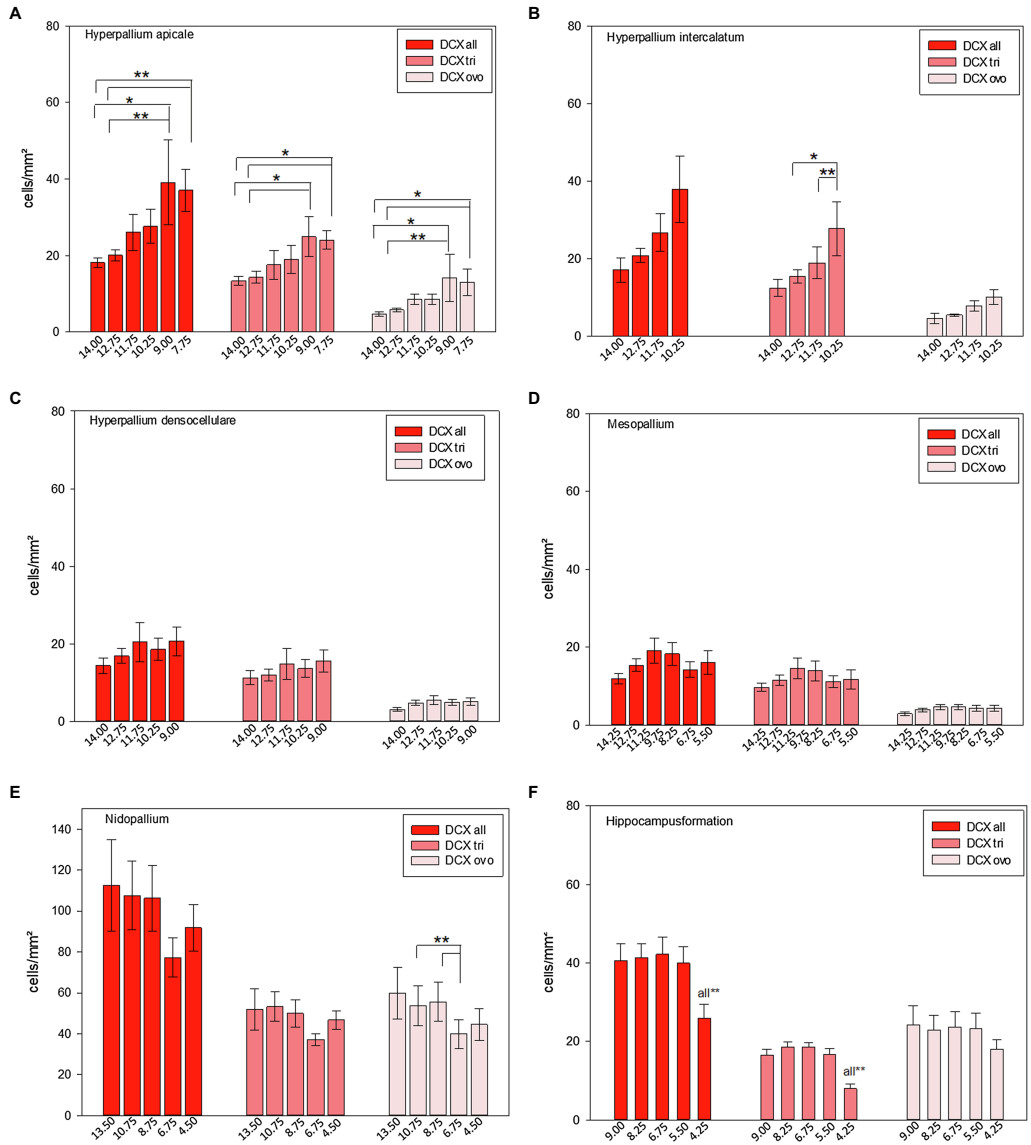
Distribution of DCX+ cells/mm^2^ of different atlas levels along the anterior–posterior axis in different regions of the pigeon (*n* = 9). **(A)** Hyperpallium apicale, **(B)** Hyperpallium intercalatum, **(C)** Hyperpallium densocellulare, **(D)** Mesopallium, **(E)** Nidopallium, **(F)** Hippocampal formation. Two different types of DCX+ cells were recognized, triangular (−tri) and ovoid (−ovo) cells. Atlas level A13.50 in nidopallium represents NF, A10.75 and A8.75 NI and A6.75 and A4.50 NC. Bars represent mean ± SE. Asterisks indicate significant differences (Wilcoxon Signed Rank Test, ^*^p < 0.05, ^**^p < 0.01).

Along the anterior–posterior axis, summarized DCX labelled cells just showed a different distribution in the apical hyperpallium [FRM ANOVA, *χ*^2^(*n* = 9, df = 5) = 14.524, *p* = 0.013, Figure 12A] and the hippocampal formation (Figure 12F, see Herold et al., 2019). Triangular-shaped DCX positive cells showed anterior–posterior variations in the apical hyperpallium [FRM ANOVA, *χ*^2^(*n* = 9, df = 5) = 14.905, *p* < 0.011, Figure 12A], the intercalated hyperpallium [FRM ANOVA, *χ*^2^(*n* = 9, df = 3) = 8.200, *p* < 0.042, Figure 12B], and the hippocampal formation (Figure 12F, see Herold et al., 2019). For ovoidal-shaped DCX positive cells we found anterior–posterior variations in the apical hyperpallium [FRM ANOVA, *χ*^2^(*n* = 9, df = 5) = 13.635, *p* = 0.018, Figure 12A], and the nidopallium in total [FRM ANOVA, *χ*^2^(*n* = 9, df = 4) = 11.100, *p* = 0.025, Figure 12E]. DCX+ cells (and both, DCX tri and DCX ovo) in HA und ovoidal-shaped cells in HI showed a clear increase from anterior to posterior with highest numbers at atlas levels A9.00 (HA) and A10.25 (HI; Figure 12A+B; Supplementary Tables S5 and S6). There are anterior–posterior variations for ovoidal-shaped DCX+ cells the nidopallium (see above), but their distribution showed no clear trends (increase or decrease) along the anterior–posterior axis (Figure 12E). More cells tend to be located in the anterior parts (A13.50–A8.75) within NF and NI and lower numbers at atlas level A 6.75 and A4.25, where NC is located (Figure 12E; Supplementary Tables S5 and S6). In the hippocampal formation, the number of DCX+ cells specifically dropped at the most posterior parts around atlas level A4.25. This effect mainly results from a decrease of triangular DCX positive cells, while ovoidal cells were almost equally distributed along the anterior–posterior axis (Figure 12F, see Herold et al., 2019). The densocellular hyperpallium and the mesopallium did not show any anterior-posterior variations (Figures 12C,D) Remaining individual values and statistical values can be found in the Supplementary Tables S5 and S6.

## Discussion

The recent study provides the first comprehensive analysis of adult neuro- and gliogenesis in the forebrain of the domestic pigeon and offers a four-stage classification for developmental markers. Our analysis revealed a high level of adult neurogenesis and plasticity and specified it. We could show for the first time that all kinds of neurogenerative cell stages were proved in the analysed brain structures. Adult newborn, proliferating, differentiating and maturing cells were widely distributed but with regional differences in their spatial distribution. Thereby, the hippocampal formation stands out with the highest numbers of newborn cells, followed by the intercalated hyperpallium. The region with the lowest numbers of newborn cells was the (intermediate) nidopallium. However, nidopallial areas, including the nidopallium caudolaterale as the functional equivalent to the mammalian prefrontal cortex, showed low neurogenesis, but the highest number of DCX+ cells. Generally, the quantity of DCX+ cells exceeded that of BrdU+ cells. In addition, the number of newborn glial cells usually exceeded the number of newborn neurons and individual structures showed variations along the anterior–posterior axis.

In the following, we will discuss at first the results of our qualitative analyses (including methodological considerations) followed by the discussion of the quantitative analysis (including functional considerations). Finally, we will discuss whether adult neurogenesis may have a specific role in spatial cognition and memory of birds.

### Different Stages of Adult Neurogenesis in the Pigeon Forebrain and Methodological Considerations

In the recent study, a number of markers that are expressed at specific stages during neuronal development or that indicate actively dividing cells have been tested in addition to BrdU. Using BrdU labelling, combined with neuronal markers, advanced research about adult neurogenesis, led to the detection of new neurons in different species and is still the “gold standard” for investigating adult neurogenesis (Eriksson et al., 1998; Kornack and Rakic, 1999; Gould et al., 1999b; Kuhn et al., 2018). In contrast to many other studies, we considered that neurons usually need several weeks to become matured and performed our brain analyses not until 12 weeks after BrdU application.

In addition to the “gold standard,” we used different markers for the qualitative analysis that have been proved to be valuable to indicate distinct stem or progenitor cells during the progression of cells to become either mature neurons or mature glia cells in different species and in different stem cell niches/regions (Kee et al., 2002; Kempermann et al., 2004; Knoth et al., 2010; Hsieh, 2012; Balthazart and Ball, 2014; Herold et al., 2019; Labusch et al., 2020; Denoth-Lippuner and Jessberger, 2021). The finding about GFAP and Sox2 co-staining in the ventricular zones along the whole anterior–posterior axis implies that adult neurogenesis occurs all over the pigeon forebrain, starting from different spots and is in line with the analysed distribution of BrdU+ cells in this study and an earlier description of proliferative spots detected with high BrdU-labelling in those regions (Melleu et al., 2013).

The next stages of neurogenesis were explored with Tbr2. Here, Tbr2+ cells were associated to stage 1, 2 and 3 (see “Results”). In mammals, Tbr2 is typically expressed in intermediate non-radial neuronal progenitors during both, developmental and adult neurogenesis (Hevner, 2019; Denoth-Lippuner and Jessberger, 2021) and supports migration of newborn cells in the hippocampus (Nelson et al., 2020). In the SVZ or SGZ of mammals, Tbr2 is expressed in so-called transit amplifying progenitors that still have the potential to develop into neurons and astrocytes, to divide and often occur in clusters during adult neurogenesis (Kempermann et al., 2004; Hsieh, 2012; Nelson et al., 2020). In addition to these clusters, Tbr2+ cells with mitotic figures were detected in the pigeon brain and considered stage 2 “cloudy” cells. It is most likely that they develop into neurons because in the developing neocortex of mammals, identically looking mitotic Tbr2+ cells have been described in the VZ, SVZ and IZ to later form neurons (Englund et al., 2005).

Further, Prox1 and DCX were used to detect subsequent stages during the maturation of adult newborn neurons (Knoth et al., 2010; Lavado et al., 2010; Kempermann et al., 2015; Denoth-Lippuner and Jessberger, 2021). Dependent on their occurrence in clusters, size and shape Prox1+ cells were sorted to stage 2 and 3 cells, while DCX+ cells were categorized to stage 3 and 4 cells. Finally, triple-labelling of DCX, CB and CR showed that all three markers can be considered stage 3 and stage 4 markers of differentially shaped cells with either low or high numbers of processes, ramifications and cell contacts, with high densities representing late integrative phases of adult neurons. Here, CB and CR were used as markers that indicate late integrative and as common markers for neurons that are typically bound to established neuronal networks in birds and mammals (Baimbridge et al., 1992; Hof et al., 1999; Heyers et al., 2008; Bruce et al., 2016). To underpin this “stage-classification,” Ki67 and DCX double labelling experiments confirmed that DCX+ multipolar (juvenile) neurons in the pigeon brain can also express Ki67 what means that they are still proliferative. Immunohistochemical visualization of cell proliferation by Ki-67 has been validated against the BrdU labelling technique elsewhere and showed high correlations between the two methods (Kee et al., 2002). However, BrdU, as we have used it (immunohistochemical processing 3 months after injection), also allowed us (in combination with NeuN) the detection and quantification of newborn adult neurons that were born 12 weeks before. That there are, even 12 weeks after BrdU administration, DCX+/BrdU+ double labelled cells in the pigeon brain might be an indication for (newborn) juvenile neurons which are in a state of rest. It is known that similar markers of developmental stages of juvenile neurons can vary enormously in their temporal correlations and expression relative to proliferating cells. For example, DCX can be also expressed in adult, non-newly generated cell populations, marking sites of neuronal plasticity *per se* (Lipp and Bonfanti, 2016). However, with our double-labelling with BrdU we can ensure that these cells are newborn.

Finally, our quantitative analysis of DCX+ and multipolar NeuN+/BrdU+ neurons confirmed that adult neurogenesis is accumulated differentially in specific brain regions.

### Adult Neurogenesis in Different Forebrain Areas of the Pigeon Brain and Functional Considerations

Previous studies already showed a high number of proliferating cells in several brain regions of the pigeon and suggested a high level of adult neurogenesis and plasticity (Melleu et al., 2013; Mazengenya et al., 2017; Herold et al., 2019). Our analysis confirmed a high level of adult neurogenesis and plasticity and specified it.

Within the hyperpallium, HI showed the highest number of newborn and proliferating cells. Additionally, there was an increase of adult newborn neurons from anterior to posterior in HI. HI is part of the so-called avian Wulst that is composed of the apical part of the hyperpallium (HA), the interstitial part of HA (IHA), the intercalated hyperpallium (HI) and the densocellular hyperpallium (HD; Shimizu and Karten, 1990; Reiner et al., 2004; Atoji et al., 2018). Functionally, the avian Wulst is divided into a small “rostral Wulst” that receives somatosensory input and the much larger caudal part that receives visual information. Our study mainly comprised data of the visual part; however, data of atlas level A14.00 included parts of the rostral situated somatosensory part. The principal projection of HI is a reciprocal connection to HA (Atoji et al., 2018). Besides, HI projects to the medial striatum and is reciprocally connected to the frontolateral nidopallium (Atoji and Wild, 2019). Thalamic input to HI mainly derives from the lateral part of the dorsolateral anterior thalamic nucleus (DLL). There are hardly interconnections between IHA, HI and HD but many fibers within them (Atoji et al., 2018; Atoji and Wild, 2019; Stacho et al., 2020). In contrast to HI, HD showed neither large amounts of newborn or immature cells, nor anterior–posterior variations. It was suggested that visual information, e.g., during migration or homing reaches not just the dorsolateral subdivision of the hippocampal formation (DL) but also HD and that HD serves as a gateway to DL (Patzke et al., 2010; Rastogi et al., 2011; Atoji and Wild, 2019). Besides, the olfactory system connects with HD and it seems likely that HD plays a dual role in visual and olfactory processing (Patzke et al., 2011; Atoji and Wild, 2014, 2019). Here, the higher level of adult neurogenesis in HI and the unremarkable number of newborn neurons and immature cells in HD indicate a greater need of plasticity in HI and, conversely, a greater need of stable conditions (“conservatism”) in HD. The occurrence of more newborn cells/neurons in the posterior part of HI suggests a functional specialization within this structure. What kind of functional specialization this is, needs to be further elucidated.

The intermediate nidopallium belongs, together with the entopallium and the ventral mesopallium, to the so-called avian visual dorsal ventricular ridge (DVR) and is characterized by a lot of efferent projections, reaching several distinct associative and premotor nidopallial areas (Fernández et al., 2019). Besides, NI is part of the visual tectofugal system in birds and also evinced projections to and from other (mesopallial) visual, auditory and sensory areas (Stacho et al., 2020). Additionally, the nidopallium intermedium medialis pars lateralis (NIML) seems to be important for multi-component behavior (Rook et al., 2021). Fernández et al. (2019) also detected reciprocal projections to the visual hyperpallium and lateral striatum originating from different cell populations within NI. There exists the assumption that the DVR forms the core of a dense network of highly specific connections between this region and other higher order areas of the avian pallium (Fernández et al., 2019). This network is part of iteratively organized canonical circuits that structurally resembles mammalian cortical organization (Stacho et al., 2020). In our study, NI showed the lowest level of newborn cells but the highest level of DCX+ cells. Apparently, for this network and the hierarchical processing during goal-directed behaviour, newborn cells (neither neurons nor glial cells) seem to be not mandatory at the current state but proliferating cells that can be added to existing networks are sufficient.

Our analysis of different atlas levels along the anterior–posterior axis showed that newborn glial cells decreased from anterior to posterior levels in the nidopallium. Regarding the different subdivisions of the nidopallium, this implicates that the frontal nidopallium exhibits more newborn glial cells than the intermediate nidopallium, while the caudal nidopallium exhibits the lowest number of newborn nidopallial glial cells. This is most likely associated to the close proximity of NF to enriched stem cell niches and the migration of proliferative cells from anterior to more posterior locations (Balthazart et al., 2008; Melleu et al., 2013; Balthazart and Ball, 2014). Parts of the frontal nidopallium participate in the avian pallial somatosensory system and belong to the trigeminal DVR (NFT, Nidopallium frontotrigeminale; Stacho et al., 2020; Fernández et al., 2021). However, we did not differentiate between NFT and the other regions of NF (e.g., frontolateral and frontomedial nidopallium). Such a differentiation might bring more clarity about the relevance of newborn glial cells in NF. The caudal nidopallium, with its subdivisions NCC (caudocentral nidopallium), NCL (caudolateral nidopallium) and NCM (caudomedial nidopallium) is a multimodal area and serves comparable functions to the mammalian prefrontal cortex (Herold et al., 2014). Thus, it would have been expected that we find a lot of newborn neurons in this region if newborn neurons play an important role for higher cognitive functions *per se*. Because newborn neurons showed low levels in NC, this may lead to the conclusion that the functional relevance of newborn neurons on cognitive abilities, at least in the NC, is modest. However, a high number of non-matured neurons and thus plasticity seems to be important.

### A Specific Role for New Neurons in Spatial Cognition and Memory of Birds?

Homing pigeons have been often used as a model to study complex cognitive processes that depend on learning, memory and choice behaviour in line with certain pallial structures. Therefore, the vast amount of behavioural and anatomical data has rendered the pigeon one of the key model species of behavioural and comparative neuroscience and to study learning theories (Shanahan et al., 2013; Güntürkün et al., 2014, 2021; Güntürkün and Bugnyar, 2016; Scarf et al., 2016). However, apart from the hippocampus, none of the studies considered adult neurogenesis in other pallial areas as a relevant factor for their astonishing cognitive abilities. Our results might indicate that there is just a small significance of adult neurogenesis for complex cognitive abilities processed in hyperpallial and nidopallial regions. However, an exception could be spatial cognition or so-called pattern separation, respectively, in the hippocampus. The hippocampal formation plays a key role in processing spatial information and showed the highest numbers of newborn and immature cells compared to the other pallial structures in our study. The high level of adult neurogenesis in the hippocampus is in line with the theory that adult hippocampal neurogenesis has, at least in mammals, a functional relevance for determining whether an experience is something that has been experienced before and whether a detected or sensed object is similar to or different from objects detected in the recent past (Gage, 2021). The more similar the objects are, the greater the burden on adult neurogenesis to make this determination. Although old memories remain, matching them with current experiences becomes difficult without adult neurogenesis (Gage, 2021). Further, anterior–posterior variations in the hippocampal formation were found for proliferative DCX+ cells, but not for NeuN+/BrdU+ or GFAP+/BrdU+ cells (Herold et al., 2019). In addition, an anterior–posterior decrease of newborn glial cells in the lateral blade of the hippocampal V-region (Vl) was detected and explained with interregional specializations within the hippocampal formation (Herold et al., 2019). Together with the finding of the nidopallium this may now be revisited and suggests that the core of newborn glia cells may hatch in anterior ventricular niche zones and therefore, accumulate in these regions as they do not need to migrate over long distances. Both findings, however, suggest a functional specialization along the anterior–posterior axis of this regions. Interestingly, in the hyperpallium and mesopallium it was found the other way around. Newborn glial cells increased from anterior to more posterior locations, which may imply that cells here derive from additional more posterior niches (shorter versus longer distance) or locally in the parenchym, which would be supported by the findings here of cell clusters in the mesopallium and hyperpallium that include progenitor cells which are still not fate-specific.

Our finding of larger and more matured compared to smaller and immature cells in the hyper- and mesopallium is partly in line with Melleu et al. (2013) who showed that the pallial regions HA and M were populated by multipolar, polygonal and large DCX+ cells, endowed with extensively branched, heavily stained dendrite-like processes. However, in contrast to Melleu et al. (2013), we cannot confirm this finding for the nidopallium. Our nidopallium data showed a balanced ratio of large triangular and small ovoidal cells or even a higher number of ovoidal cells. Additionally, the authors observed a particularly dense concentration of DCX+ cells in the caudolateral and caudoventral aspects of the nidopallium. We also found high numbers of immature cells in the nidopallium, but in NF and particularly in NI, not in NC. One explanation for this discrepancy could be, that, Melleu et al. (2013) just described the distribution of proliferating cells in a qualitative way and focused on the ventricular zone as a proliferative “hotspot.” Another explanation could be the different origin of the investigated pigeons. In their study, the authors examined pigeons which were raised and lived permanently in a loft. Our examined pigeons had the possibility to fly around the loft and thus, to gain navigational experience and to perceive their whole environment. Both, spatial experience and an enriched environment have a positive effect on adult neurogenesis and can lead to more proliferating cells, at least in the hippocampus (Patel et al., 1997; Melleu et al., 2016). Further it was reported that in the most telencephalic regions, the density of PCNA- and DCX-immunoreactive cells increased from rostral to caudal, except in the mesopallium where the density decreased from rostral to middle levels and then increased caudally (Mazengenya et al., 2017). For HA and HI we can confirm this results, for HD and M the situation is not so clear. One explanation could be again that we compared here quantitative data in our study with the qualitative data from Mazengenya et al. (2017).

The actual number of adult-generated neurons is a small proportion of the total population of neurons and as well of the total number of newly generated cells. For identifying newborn neurons, we used NeuN in combination with BrdU labelling and although it is possible, that NeuN does not stain all neuronal types, it is a reliable and approved marker for mature neurons (Mullen et al., 1992; Gross, 2000). We suggested that the main function of these newborn neurons is not the compensation of declined neurons but that they are essential for the rapid adaptation to environmental changes. Besides, the existence of adult-generated neurons in the hippocampus and elsewhere, and the possibility that these cells may function in learning and memory can offer new mechanisms for information storage in the brain. It may be that learning and memory involve the development of entirely new circuits with new and previously unused elements as well as the modulation of older circuits and connections (Clelland et al., 2009; Christian et al., 2014; Denoth-Lippuner and Jessberger, 2021). This could be also an explanation for our finding that the hippocampal formation showed more immature ovoidal-shaped DCX+ cells compared to more matured triangular DCX+ cells, whereas the hyperpallial structures, the mesopallium and the nidopallium showed more matured triangular DCX+ cells.

Interestingly, in pigeons Meskenaite et al. (2016) showed a typical decline of proliferating cells during the first year of life, but a higher level of new neurons in the triangular area of the hippocampus in aged pigeons. In their study, generally, the hippocampus showed a decline of neurogenesis, but to a lesser extent compared to other regions. However, in the triangular area of the hippocampus, the oldest birds (up to 14 years) showed nearly twice the number of neurons as compared to young adult pigeons. Their findings suggests that the increased numbers might reflect navigational experience and, possibly, expanded spatial memory (Meskenaite et al., 2016). It further confirms our assumption that adult neurogenesis likely has different functions for spatial cognition compared to higher cognitive functions in general. Although their study just examined the olfactory bulb and the hippocampal formation, it was the first example that aging does not necessarily result in reduced number of neurons. It indicates that the precise role of new neurons in the adult (or aged) brain is region-specific and remains to be determined. This highlights the importance of future studies addressing the impact of different factors on adult neurogenesis and, even more important, on brain function. Further, most of these studies just considered the hippocampus and did not clarify the consequences of reduced or increased adult neurogenesis on brain function. Melleu et al. (2016) at least suggested that enriched environment (EE) favored behavioral inhibition and neurogenesis in pigeons. Pigeons housed under EE exhibited more DCX+ cells in the hippocampus and fewer DCX+ cells in the lateral striatum than those housed in a standard environment. Additionally, pigeons housed in EE responded with higher immobility when confronted with a novel object and longer duration in a tonic immobility test (Melleu et al., 2016).

A high number of immature neurons was also shown in the dentate gyrus of neurologically healthy humans (Moreno-Jiménez et al., 2019). Interestingly, the number and maturation of these neurons progressively declined during Alzheimer’s disease what provides evidence for impaired neurogenesis as a potentially relevant mechanism underlying memory deficits in Alzheimer’s disease (Moreno-Jiménez et al., 2019). Terreros-Roncal et al. (2021) also showed a contribution of adult hippocampal neurogenesis to amyotrophic lateral sclerosis (ALS), Huntington’s disease, Parkinson’s disease and dementia. Although the proportion of newborn neurons is a minor population compared to the total number of neurons, and although there are skeptics about the involvement of adult neurogenesis in neuropsychatric diseases (Duque et al., 2021), the continuous addition of new neurons over a lifetime implies that adult neurogenesis could add substantial structural and functional plasticity at least in the hippocampus of humans (Toda and Gage, 2018). However, of course it has to be considered that translating rodent and human findings to the brain of homing pigeons and vice versa should be done with caution. There are different views on the functional impact of adult neurogenesis (Lipp and Bonfanti, 2016) and studies that do not fit the picture (Amrein et al., 2007; Patzke et al., 2015). From a comparative point of view, adult neurogenesis in mammals differs at least from adult neurogenesis as found in bird species with seasonal variation in behavior-controlling brain structures (Nottebohm, 2004). Generally, adult neurogenesis appears as protracted juvenile neurogenesis that is downregulated differentially in various brain structures depending on species and age levels (Lipp and Bonfanti, 2016).

Since the first evidence of adult neurogenesis in higher vertebrates (Altman, 1962, 1969; Altman and Das, 1965), many studies have been conducted, particularly about adult hippocampal neurogenesis in mammals, but also in other species including birds. However, there are still many open questions and the species-specific functional relevance of adult neurogenesis in individual brain regions is yet not clarified because different parameters are related to adult neurogenesis. Toda and Gage (2018) even argued that the regulation of adult neurogenesis by individual’s behaviour, experience and emotional/biological status is one of the intriguing and fundamental features of adult neurogenesis. They postulated an on-demand neurogenesis in response to physiological and environmental signals which would provide an additional layer of plasticity. Lipp and Bonfanti (2016) postulated that the field of adult neurogenesis has a solid basis for understanding the molecular and developmental mechanisms regulating neurogenesis but that the functional consequences for regeneration and behavioural/cognitive effects is still elusive and contradictory. In our opinion, the regulation of adult neurogenesis by environmental and biological factors can occur in both, positive and negative directions, and consequently can influence brain function. Thus, it is a challenge of future studies to investigate and specify how environmental or internal stimuli regulate and influence adult neurogenesis and how newborn neurons contribute to brain function. Here, comparing adult neurogenesis of pigeons with different levels of navigational experience or newly acquired experiences might be of interest. Also, comparisons between homing pigeons and non-homing pigeon breeds can provide more insight into this topic.

In conclusion, our study provides a fundamental basis for future studies in the avian brain and may facilitate the interpretation and comparison to other vertebrates in the future. The influences on and the impact of adult neurogenesis likely varies among different species and strains. This point should be carefully taken into account when comparing different studies. Adult neurogenesis is ubiquitous in the pigeon forebrain, but we revealed not only regional differences of the level of adult neurogenesis, but also intraregional differences along the anterior–posterior axis and different cell types. This, as well as the possibility of newly generated inter- and intraregional connections and cell types have to be considered in all further investigations.

## Data Availability Statement

The original contributions presented in the study are included in the article/Supplementary Material, further inquiries can be directed to the corresponding author.

## Ethics Statement

The animal study was reviewed and approved by Committee on the Ethics of Animal Experiments of the state of North Rhine-Westphalia, Germany (Ref. 84-02.04.2014.A345).

## Author Contributions

JM and CH contributed to study concept, design, data acquisition, analysis, and interpretation of data. JM and CH contributed to drafting of the manuscript and preparing the figures. NN and KL data acquisition. NN, KL, SC, and KA critical reading and commenting on the final version of the manuscript. All authors contributed to the article and approved the submitted version.

## Conflict of Interest

The authors declare that the research was conducted in the absence of any commercial or financial relationships that could be construed as a potential conflict of interest.

## Publisher’s Note

All claims expressed in this article are solely those of the authors and do not necessarily represent those of their affiliated organizations, or those of the publisher, the editors and the reviewers. Any product that may be evaluated in this article, or claim that may be made by its manufacturer, is not guaranteed or endorsed by the publisher.

## Abbreviations

Arco: arcopallium
BrdU: 5-bromo-deoxyuridine
CB: Calbindin
CDL: Area corticoidea lateralis
CoS: Nucleus commissura septi
CPi: Cortex piriformis
CR: Calretinin
DCX: doublecortin
DL: dorsolateral region of HF
DLv: ventral dorsolateral region of HF
DMd: dorsal dorsomedial region of HF
DMv: ventral dorsomedial region of HF
DVR: dorsal ventricular ridge
GFAP: glial fibrillary acidic protein
FITC: Fluorescein isothyocyanate
H: hyperpallium
HA: apical hyperpallium
HD: densocellular hyperpallium
HF: hippocampal formation
HI: intercalated hyperpallium
HVC: high vocal centre
IHA: interstitial part of HA
LSt: lateral striatum
MSt: medial striatum
M: mesopallium
NeuN: neural nuclei
N: nidopallium
NC: caudal nidopallium
NCC: caudocentral nidopallium
NCL: caudolateral nidopallium
NCM: caudomedial nidopallium
NI: intermediate nidopallium
NIML: Nidopallium intermedium medialis pars lateralis
NF: frontal nidopallium
NFT: frontotrigeminal nidopallium
PoAb: posterior amygdala pars basalis
OB: olfactory bulb
ovo: ovoidal DCX+ cell
PBS: phosphate buffered saline
Prox1: Prospero Homeobox 1 protein
SM: Nucleus septalis medialis
SL: Nucleus septalis lateralis
S100ß: S100 calcium binding protein ß
SVZ: subventricular zone
Tbr2: T-box brain protein 2
TnA: Nucleus taenia of the amygdala
Tr: triangular area of HF
tri: triangular DCX + cell
TuO: olfactory tubercle
V: ventricle
Vl: lateral part of the hippocampal V-region
Vm: medial part of the hippocampal V-region
VZ: ventricular zone.
