# Supplementary material for: Regional Patterning of Adult Neurogenesis in the Homing Pigeon’s Brain
Source: Front Psychol. 2022 Jul 8;13:889001. doi: 10.3389/fpsyg.2022.889001 (PMC9311432; doi:10.3389/fpsyg.2022.889001)
Supplement: Supplementary file 1 [file Table_1.DOCX]

Supplementary Material

# 1. Supplementary Figures and Tables

## 1.1. Supplementary Figures


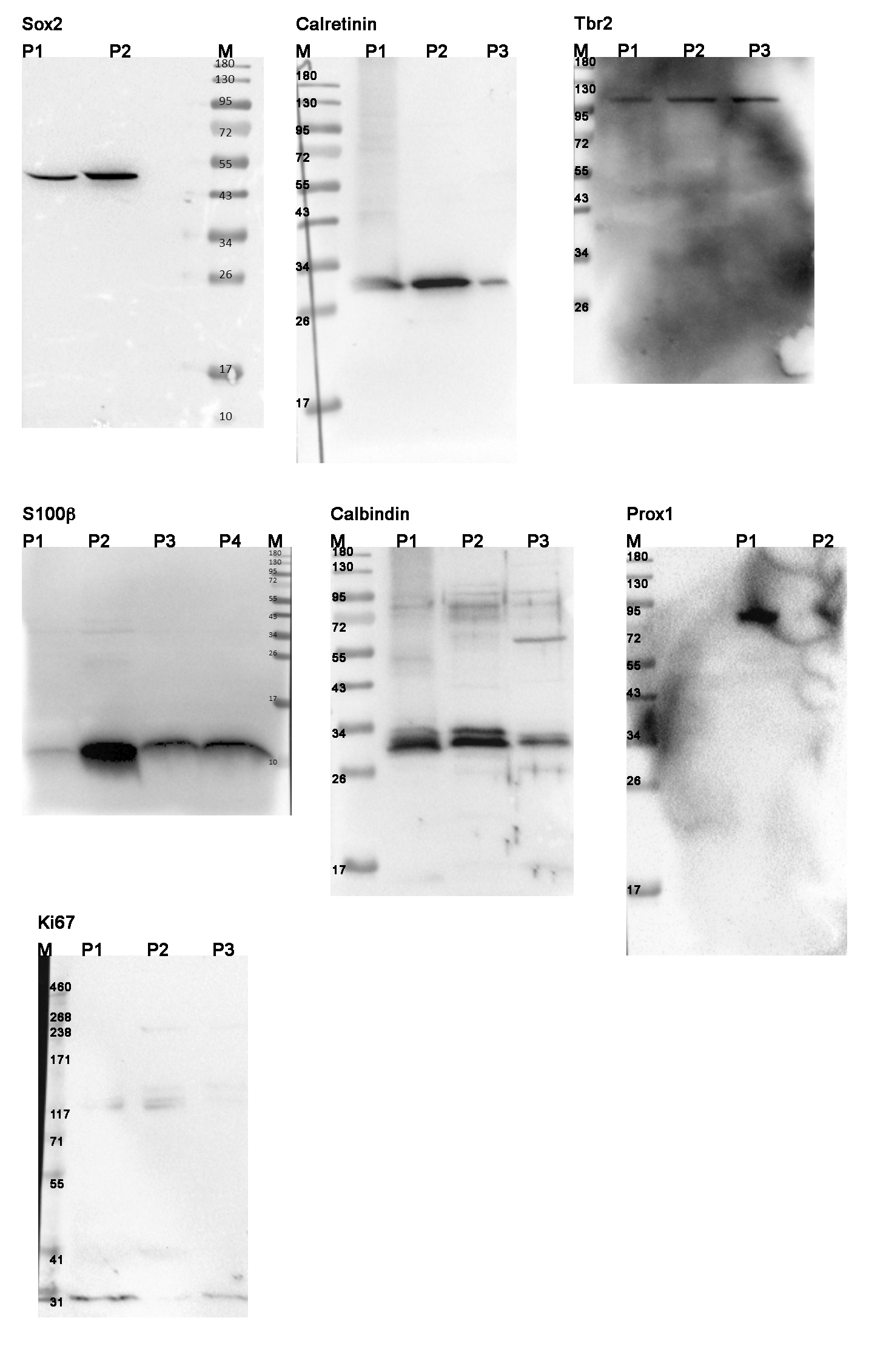


Fig. S1 Western blot controls of antibody binding with different forebrain probes.


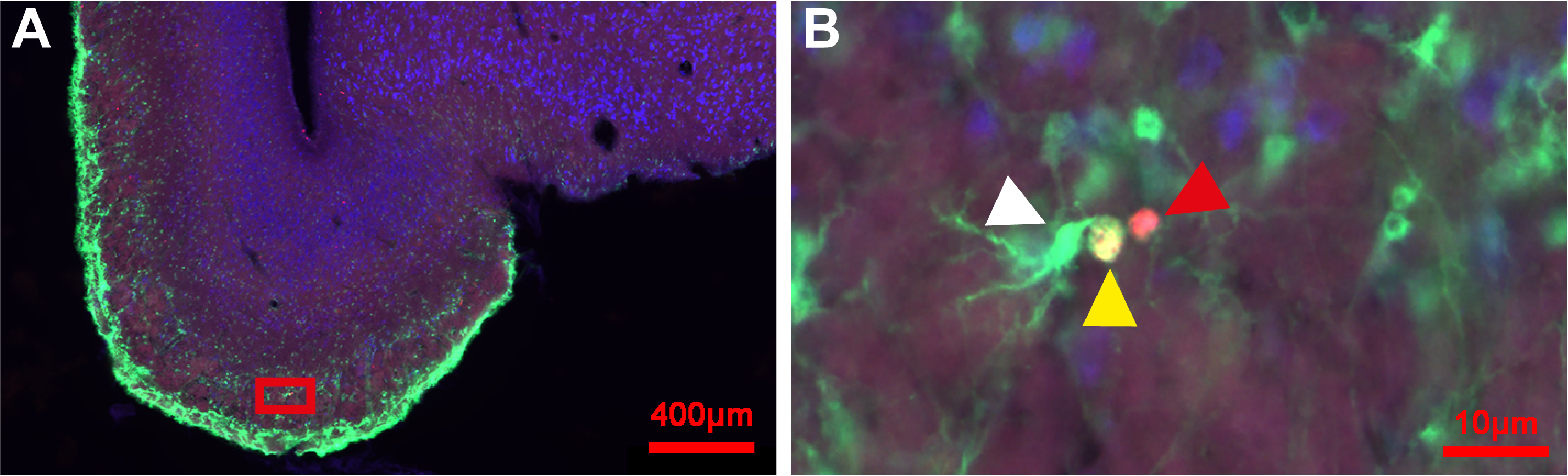


Fig. S2. OB of the adult pigeon immunostained for S100b, NeuN and BrdU at atlas level A13.75. (A) S100b+(green), NeuN+(dark blue) and BrdU+(red) cell distribution in the OB. (B) Stem cell niche figure in the external plexiform and mitral cell layer showing a S100b+ (green) cell with astral-like fibres and a typical short endfoot process (white arrow) in close proximity to a S100b+/BrdU+ double labelled (yellow) cell and BrdU+ (red) cell without further staining for S100b or NeuN.


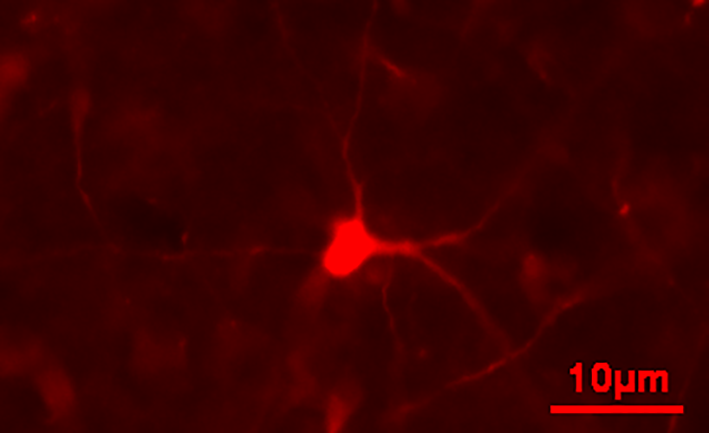


Fig. S3 DCX+ rounded basket cell in the nidopallium at atlas level A11.25.

## 1.2. Supplementary Tables

Table S1

**b**

Statistical results of comparisons between BrdU, BrdU+, BrdU+/NeuN+ and BrdU+/GFAP+ cells/mm^2^ in different telencephalic regions of the pigeon (Wilcoxon Signed Rank Test).

| **BrdU total** | HA | HI | HD | M | NF | NI | NC | HF |
| --- | --- | --- | --- | --- | --- | --- | --- | --- |
| HA |  | **T=43.00**  **Z=2.55** | T=17.00  Z=1.01 | T=3.00  Z=0.18 | T=-9.00  Z=-0.53 | **T=-41.00**  **Z=-2.43** | **T=-45.00**  **Z=-2.67** | T=31.00  Z=1.84 |
|  |  | **p=0.008** | p=0.359 | p=0.910 | p=0.652 | **p=0.012** | **p=0.004** | p=0.074 |
| HI |  |  | **T=-45.00**  **Z=-2.67** | **T=-35.00**  **Z=-2.08** | T=-27.00  Z=-1.60 | **T=-41.00**  **Z=-2.43** | **T=-45.00**  **Z=-2.67** | T=13.00  Z=0.77 |
|  |  |  | **p=0.004** | **p=0.039** | p=0.129 | **p=0.012** | **p=0.004** | p=0.496 |
| HD |  |  |  | T=-5.00  Z=-0.30 | T=-15.00  Z=-0.89 | **T=-39.00**  **Z=-2.31** | **T=-45.00**  **Z=-2.67** | T=-31.00  Z=-1.84 |
|  |  |  |  | p=0.820 | p=0.426 | **p=0.020** | **p=0.004** | p=0.074 |
| M |  |  |  |  | T=-13.00  Z=-0.77 | **T=-39.00**  **Z=-2.31** | **T=-45.00**  **Z=-2.67** | T=33.00  Z=1.96 |
|  |  |  |  |  | p=0.496 | **p=0.020** | **p=0.004** | p=0.055 |
| NF |  |  |  |  |  | **T=-39.00**  **Z=-2.32** | **T=-41.00**  **Z=-2.43** | **T=-45.00**  **Z=-2.67** |
|  |  |  |  |  |  | **p=0.020** | **p=0.012** | **p=0.004** |
| NI |  |  |  |  |  |  | **T=43.00**  **Z=2.55** | **T=-45.00**  **Z=-2.67** |
|  |  |  |  |  |  |  | **p=0.008** | **p=0.004** |
| NC |  |  |  |  |  |  |  | **T=-45.00**  **Z=-2.67** |
|  |  |  |  |  |  |  |  | **p=0.004** |
| HF |  |  |  |  |  |  |  |  |
|  |  |  |  |  |  |  |  |  |
|  |  |  |  |  |  |  |  |  |
| **BrdU+** | HA | HI | HD | M | NF | NI | NC | HF |
| HA |  | T=23.00  Z=1.36 | T=3.00  Z=0.18 | T=6.00  Z=0.36 | **T=-37.00**  **Z=-2.12** | **T=-45.00**  **Z=-2.67** | **T=-45.00**  **Z=-2.67** | T=-17  Z=-1.01 |
|  |  | p=0.203 | p=0.910 | p=0.734 | **p=0.027** | **p=0.009** | **p=0.004** | p=0.359 |
| HI |  |  | T=-33.00  Z=-1.96 | T=-27.00  Z=-1.56 | **T=-41.00**  **Z=2.43** | **T=-43.00**  **Z=-2.55** | **T=45.00**  **Z=2.67** | T=23.00  Z=1.36 |
|  |  |  | p=0.055 | p=0.129 | **p=0.012** | **p=0.008** | **p=0.004** | p=0.203 |
| HD |  |  |  | T=15.00  Z=0.89 | **T=-37.00**  **Z=-2.12** | **T=-43.00**  **Z=-2.55** | **T=45.00**  **Z=2.67** | T=5.00  Z=0.30 |
|  |  |  |  | p=0.426 | **p=0.027** | **p=0.008** | **p=0.004** | p=0.820 |
| M |  |  |  |  | **T=-37.00**  **Z=-2.12** | **T=-45.00**  **Z=-2.67** | **T=45.00**  **Z=2.67** | T=13.00  Z=0.77 |
|  |  |  |  |  | **p=0.027** | **p=0.009** | **p=0.004** | p=0.496 |
| NF |  |  |  |  |  | **T=-41.00**  **Z=-2.43** | T=-7.00  Z=-0.42 | **T=-39.00**  **Z=-2.32** |
|  |  |  |  |  |  | **p=0.012** | p=0.734 | **p=0.020** |
| NI |  |  |  |  |  |  | T=25.00  Z=1.48 | **T=-45.00**  **Z=-2.67** |
|  |  |  |  |  |  |  | p=0.164 | **p=0.004** |
| NC |  |  |  |  |  |  |  | T=-20.00  Z=-1.24 |
|  |  |  |  |  |  |  |  | p=0.098 |
| HF |  |  |  |  |  |  |  |  |
|  |  |  |  |  |  |  |  |  |
|  |  |  |  |  |  |  |  |  |
|  |  |  |  |  |  |  |  |  |
| **BrdU+/NeuN+** | HA | HI | HD | M | NF | NI | NC | HF |
| HA |  | **T=37.00**  **Z=2.19** | T=31.00  Z=1.84 | T=15.00  Z=0.89 | **T=-45.00**  **Z=-2.67** | **T=45.00**  **Z=2.67** | T=-17.00  Z=-1.01 | T=-11.00  Z=-0.65 |
|  |  | **p=0.027** | p=0.074 | p=0.426 | **p=0.004** | **p=0.004** | p=0.359 | p=0.570 |
| HI |  |  | **T=-37.00**  **Z=-2.19** | **T=-39.00**  **Z=-2.31** | **T=-45.00**  **Z=-2.67** | **T=45.00**  **Z=2.67** | **T=-35.00**  **Z=-2.07** | T=13.00  Z=0.77 |
|  |  |  | **p=0.027** | **p=0.020** | **p=0.004** | **p=0.004** | **p=0.039** | p=0.496 |
| HD |  |  |  | T=-15.00  Z=-0.89 | **T=-45.00**  **Z=-2.67** | **T=45.00**  **Z=2.67** | T=-27.00  Z=-1.60 | T=3.00  Z=0.18 |
|  |  |  |  | p=0.426 | **p=0.004** | **p=0.004** | p=0.129 | p=0.910 |
| M |  |  |  |  | **T=-43.00**  **Z=-2.55** | **T=45.00**  **Z=2.67** | T=-31.00  Z=-1.84 | T=-3.00  Z=-0.18 |
|  |  |  |  |  | **p=0.008** | **p=0.004** | p=0.074 | p=0.910 |
| NF |  |  |  |  |  | T=-9.00  Z=-0.53 | T=15.00  Z=0.89 | T=25.00  Z=1.48 |
|  |  |  |  |  |  | p=0.652 | p=0.426 | p=0.164 |
| NI |  |  |  |  |  |  | **T=35.00**  **Z=2.07** | T=31.00  Z=1.84 |
|  |  |  |  |  |  |  | **p=0.039** | p=0.074 |
| NC |  |  |  |  |  |  |  | T=-19.00  Z=-1.13 |
|  |  |  |  |  |  |  |  | p=0.301 |
| HF |  |  |  |  |  |  |  |  |
|  |  |  |  |  |  |  |  |  |
|  |  |  |  |  |  |  |  |  |
|  |  |  |  |  |  |  |  |  |
| **BrdU+/GFAP+** | HA | HI | HD | M | NF | NI | NC | HF |
| HA |  | **T=32.00**  **Z=2.24** | T=21.00  Z=1.24 | T=-7.00  Z=-0.42 | T=11.00  Z=0.65 | T=19.00  Z=1.13 | **T=-45.00**  **Z=-2.67** | T=-33.00  Z=-1.96 |
|  |  | **p=0.023** | p=0.250 | p=0.734 | p=0.570 | p=0.301 | **p=0.004** | p=0.055 |
| HI |  |  | **T=-39.00**  **Z=-2.31** | T=-29.00  Z=-1.72 | T=-7.00  Z=-0.42 | T=27.00  Z=1.60 | **T=-45.00**  **Z=-2.67** | T=-23.00  Z=-1.36 |
|  |  |  | **p=0.020** | p=0.098 | p=0.734 | p=0.129 | **p=0.004** | p=0.203 |
| HD |  |  |  | T=-21.00  Z=-1.24 | T=7.00  Z=0.42 | T=21.00  Z=-1.24 | **T=-45.00**  **Z=-2.67** | T=-29.00  Z=-1.72 |
|  |  |  |  | p=0.250 | p=0.734 | p=0.250 | **p=0.004** | p=0.098 |
| M |  |  |  |  | T=11.00  Z=0.65 | T=17.00  Z=1.01 | **T=-45.00**  **Z=-2.67** | T=-33.00  Z=-1.96 |
|  |  |  |  |  | p=0.570 | p=0.359 | **p=0.004** | p=0.055 |
| NF |  |  |  |  |  | **T=-39.00**  **Z=-2.31** | **T=-45.00**  **Z=-2.67** | T=31.00  Z=1.84 |
|  |  |  |  |  |  | **p=0.020** | **p=0.004** | p=0.074 |
| NI |  |  |  |  |  |  | **T=-45.00**  **Z=-2.67** | **T=45.00**  **Z=2.67** |
|  |  |  |  |  |  |  | **p=0.004** | **p=0.004** |
| NC |  |  |  |  |  |  |  | **T=-45.00**  **Z=-2.67** |
|  |  |  |  |  |  |  |  | **p=0.004** |
| HF |  |  |  |  |  |  |  |  |
|  |  |  |  |  |  |  |  |  |

Table S2

Means±S.E. of BrdU+, BrdU+/NeuN+ and BrdU+/GFAP+ cells/mm^2^ of different atlas levels along the anterior-posterior axis in different telencephalic regions of the pigeon

| HA | BrdU total | BrdU+ | BrdU+/NeuN+ | BrdU+/GFAP+ |
| --- | --- | --- | --- | --- |
| HA |  |  |  |  |
| A14.00 | 13.85±3.15 | 4.64±2.44 | 1.83±0.71 | 7.37±1.54 |
| A 12.75 | 10.36±1.28 | 3.29±0.81 | 1.67±0.53 | 5.39±0.82 |
| A 11.50 | 9.84±1.13 | 2.87±0.41 | 1.74±0.43 | 5.24±1.19 |
| A 10.25 | 9.95±1.13 | 3.75±0.87 | 1.81±0.40 | 4.38±0.75 |
| A 9.00 | 13.33±3.64 | 6.35±2.97 | 2.35±0.76 | 4.64±0.71 |
| A 7.75 | 16.12±3.12 | 6.76±2.24 | 2.30±0.38 | 7.06±1.49 |
| HI |  |  |  |  |
| A14.00 | 14.11±3.45 | 4.70±2.59 | 1.66±0.71 | 7.76±1.78 |
| A 12.75 | 13.20±1.53 | 3.57±0.62 | 2.32±0.68 | 7.31±1.17 |
| A 11.50 | 13.79±1.60 | 4.11±0.73 | 2.77±0.75 | 6.91±1.30 |
| A 10.25 | 15.43±2.11 | 5.12±1.02 | 3.77±0.83 | 6.55±1.56 |
| A 9.00 | 23.38±3.18 | 6.39±0.91 | 6.82±2.10 | 10.16±2.27 |
| A 7.75 |  |  |  |  |
| HD |  |  |  |  |
| A14.00 | 13.68±1.94 | 4.20±0.84 | 2.03±0.63 | 7.45±1.94 |
| A 12.75 | 11.71±1.36 | 3.14±0.49 | 1.92±0.56 | 6.64±1.36 |
| A 11.50 | 10.87±1.48 | 2.98±0.52 | 2.37±0.68 | 5.51±1.48 |
| A 10.25 | 12.36±1.80 | 4.31±0.97 | 2.61±0.85 | 5.44±1.80 |
| A 9.00 | 13.57±2.18 | 5.42±1.05 | 2.90±0.65 | 5.25±2.18 |
| A 7.75 | 18.19±3.70 | 7.70±1.88 | 3.76±0.77 | 6.73±3.70 |
| M |  |  |  |  |
| A 14.25 | 12.71±2.25 | 4.65±1.44 | 2.59±0.87 | 5.46±1.25 |
| A 12.75 | 10.79±1.35 | 3.45±0.67 | 2.30±0.67 | 5.05±0.76 |
| A 11.25 | 9.98±1.43 | 3.33±0.54 | 2.10±0.47 | 4.45±1.51 |
| A 9.75 | 9.95±1.52 | 3.74±0.80 | 2.13±0.58 | 4.081.07 |
| A 8.25 | 11.17±1.99 | 4.78±1.22 | 2.41±0.75 | 3.981.16 |
| A 6.75 | 16.05±2.99 | 6.80±1.56 | 2.54±0.38 | 6.70±1.82 |
| A 5.50 | 23.40±4.35 | 8.56±2.27 | 4.53±1.32 | 10.31±2.03 |
| N |  |  |  |  |
| A 13.50 | 10.10±1.73 | 1.96±0.42 | 0.91±0.29 | 7.23±1.41 |
| A 10.75 | 5.85±0.74 | 1.15±0.23 | 0.65±0.13 | 4.05±0.62 |
| A 8.75 | 5.31±0.84 | 0.94±0.25 | 0.91±0.19 | 3.47±0.69 |
| A 6.75 | 4.17±0.52 | 1.21±0.31 | 1.25±0.32 | 1.71±0.29 |
| A 4.50 | 3.79±0.65 | 1.77±0.35 | 1.31±0.35 | 0.71±0.12 |
| HF |  |  |  |  |
| A9.50 | 14.68±3.41 | 1.96±0.67 | 1.27±0.36 | 11.45±3.18 |
| A8.25 | 15.54±2.48 | 2.78±1.81 | 1.69±0.30 | 11.07±2.26 |
| A6.75 | 13.84±1.72 | 3.28±0.75 | 2.20±0.45 | 8.35±1.66 |
| A5.50 | 16.30±3.12 | 2.20±0.22 | 2.57±0.52 | 11.53±3.13 |
| A4.25 | 14.81±2.28 | 3.24±1.13 | 1.60±0.27 | 9.97±2.27 |

Table S3

Statistical results of comparisons between BrdU, BrdU+, BrdU+/NeuN+ and BrdU+/GFAP+ cells/mm^2^ of different atlas levels and in different telencephalic regions of the pigeon (Wilcoxon Signed Rank Test).

| **HA BrdU+** | A 14.00 | A 12.75 | A 11.50 | A 10.25 | A 9.00 | A 7.75 |  |
| --- | --- | --- | --- | --- | --- | --- | --- |
| A 14.00 |  | T=3.00  Z=0.91 | T=7.00  Z=0.42 | T=11.00  Z=0.65 | T=31.00  Z=1.84 | T=33.00  Z=1.96 |  |
|  |  | p=0.910 | p=0.734 | p=0.570 | p=0.074 | p=0.055 |  |
| A 12.75 |  |  | T=-11.00  Z=-0.65 | T=27.00  Z=1.60 | T=25.00  Z=1.48 | **T=45.00**  **Z=2.67** |  |
|  |  |  | p=0.570 | p=0.129 | p=0.164 | **p=0.004** |  |
| A 11.50 |  |  |  | T=27.00  Z=1.60 | T=21.00  Z=1.24 | **T=45.00**  **Z=2.67** |  |
|  |  |  |  | p=0.129 | p=0.250 | **p=0.004** |  |
| A 10.25 |  |  |  |  | T=-31.00  Z=-1.84 | **T=43.00**  **Z=2.55** |  |
|  |  |  |  |  | p=0.074 | **p=0.008** |  |
| A 9.00 |  |  |  |  |  | T=11.00  Z=0.65 |  |
|  |  |  |  |  |  | p=0.570 |  |
| A 7.75 |  |  |  |  |  |  |  |
|  |  |  |  |  |  |  |  |
|  |  |  |  |  |  |  |  |
|  |  |  |  |  |  |  |  |
| **HA BrdU+/GFAP+** | A 14.00 | A 12.75 | A 11.50 | A 10.25 | A 9.00 | A 7.75 |  |
| A 14.00 |  | T=-31.00  Z=-1.84 | T=-27.00  Z=-1.60 | **T=-43.00**  **Z=-2.55** | T=-27.00  Z=-1.60 | T=1.00  Z=0.06 |  |
|  |  | p=0.074 | p=0.129 | **p=0.008** | p=0.129 | p=1.000 |  |
| A 12.75 |  |  | T=-25.00  Z=-1.48 | T=-31.00  Z=-1.84 | T=-15.00  Z=-0.89 | T=15.00  Z=0.089 |  |
|  |  |  | p=0.164 | p=0.074 | p=0.426 | p=0.426 |  |
| A 11.50 |  |  |  | T=-17.00  Z=-1.01 | T=-3.00  Z=-0.18 | T=29.00  Z=1.72 |  |
|  |  |  |  | p=0.359 | p=0.910 | p=0.098 |  |
| A 10.25 |  |  |  |  | T=9.00  Z=0.53 | **T=39.00**  **Z=2.31** |  |
|  |  |  |  |  | p=0.652 | **p=0.020** |  |
| A 9.00 |  |  |  |  |  | **T=39.00**  **Z=2.31** |  |
|  |  |  |  |  |  | **p=0.020** |  |
| A 7.75 |  |  |  |  |  |  |  |
|  |  |  |  |  |  |  |  |
|  |  |  |  |  |  |  |  |
|  |  |  |  |  |  |  |  |
| **HI BrdU+** | A 14.00 | A 12.75 | A 11.50 | A 10.25 | A 9.00 |  |  |
| A 14.00 |  | T=5.00  Z=0.30 | T=9.00  Z=0.53 | T=17.00  Z=1.01 | T=25.00  Z=1.48 |  |  |
|  |  | p=0.820 | p=0.652 | p=0.359 | p=0.164 |  |  |
| A 12.75 |  |  | T=17.00  Z=1.01 | **T=35.00**  **Z=2.07** | **T=41.00**  **Z=2.43** |  |  |
|  |  |  | p=0.359 | **p=0.039** | **p=0.012** |  |  |
| A 11.50 |  |  |  | T=21.00  Z=1.24 | **T=39.00**  **Z=2.31** |  |  |
|  |  |  |  | p=0.250 | **p=0.020** |  |  |
| A 10.25 |  |  |  |  | T=25.00  Z=1.48 |  |  |
|  |  |  |  |  | p=0.164 |  |  |
| A 9.00 |  |  |  |  |  |  |  |
|  |  |  |  |  |  |  |  |
|  |  |  |  |  |  |  |  |
|  |  |  |  |  |  |  |  |
| **HI BrdU+/NeuN+** | A 14.00 | A 12.75 | A 11.50 | A 10.25 | A 9.00 |  |  |
| A 14.00 |  | T=15.00  Z=0.89 | T=25.00  Z=1.48 | **T=41.00**  **Z=2.43** | **T=43.00**  **Z=2.55** |  |  |
|  |  | p=0.426 | p=0.164 | **p=0.012** | **p=0.008** |  |  |
| A 12.75 |  |  | T=15.00  Z=0.89 | **T=43.00**  **Z=2.55** | **T=45.00**  **Z=2.67** |  |  |
|  |  |  | p=0.426 | **p=0.008** | **p=0.004** |  |  |
| A 11.50 |  |  |  | **T=37.00**  **Z=2.19** | **T=43.00**  **Z=2.55** |  |  |
|  |  |  |  | **p=0.027** | **p=0.008** |  |  |
| A 10.25 |  |  |  |  | **T=39.00**  **Z=2.31** |  |  |
|  |  |  |  |  | **p=0.020** |  |  |
| A 9.00 |  |  |  |  |  |  |  |
|  |  |  |  |  |  |  |  |
|  |  |  |  |  |  |  |  |
|  |  |  |  |  |  |  |  |
| **M BrdU+** | A 14.25 | A 12.75 | A 11.25 | A 9.75 | A 8.25 | A 6.75 | A 5.50 |
| A 14.25 |  | T=-5.00  Z=-0.30 | T=-5.00  Z=-0.30 | T=-5.00  Z=-0.30 | T=7.00  Z=0.42 | T=27.00  Z=1.60 | T=-33.00  Z=-1,96 |
|  |  | p=0.820 | p=0.820 | p=0.820 | p=0.734 | p=0.129 | p=0.055 |
| A 12.75 |  |  | T=-7.00  Z=-0.42 | T=15.00  Z=0.89 | T=25.00  Z=1.48 | **T=39.00**  **Z=2.31** | **T=43.00**  **Z=2.55** |
|  |  |  | p=0.734 | p=0.426 | p=0.164 | **p=0.020** | **p=0.008** |
| A 11.25 |  |  |  | T=9.00  Z=0.53 | T=23.00  Z=1.36 | **T=41.00**  **Z=2.43** | **T=43.00**  **Z=2.55** |
|  |  |  |  | p=0.652 | p=0.203 | **p=0.012** | **p=0.008** |
| A 9.75 |  |  |  |  | T=25.00  Z=1.48 | **T=39.00**  **Z=2.31** | **T=-43.00**  **Z=2.55** |
|  |  |  |  |  | p=0.164 | **p=0.020** | **p=0.008** |
| A 8.25 |  |  |  |  |  | **T=41.00**  **Z=2.43** | **T=37.00**  **Z=2.19** |
|  |  |  |  |  |  | **p=0.012** | **p=0.027** |
| A 6.75 |  |  |  |  |  |  | T=-21.00  Z=-1.24 |
|  |  |  |  |  |  |  | p=0.250 |
| A 5.50 |  |  |  |  |  |  |  |
|  |  |  |  |  |  |  |  |
|  |  |  |  |  |  |  |  |
|  |  |  |  |  |  |  |  |
| **M BrdU+/GFAP+** | A 14.25 | A 12.75 | A 11.25 | A 9.75 | A 8.25 | A 6.75 | A 5.50 |
| A 14.25 |  | T=-1.00  Z=-0.06 | T=3.00  Z=0.18 | T=-9.00  Z=-0.53 | T=-11.00  Z=-0.65 | T=23.00  Z=1.36 | **T=-41.00**  **Z=-2.43** |
|  |  | p=1.000 | p=0.910 | p=0.652 | p=0.570 | p=0.203 | **p=0.012** |
| A 12.75 |  |  | T=-11.00  Z=-0.65 | T=-17.00  Z=-1.01 | T=-13.00  Z=-0.77 | T=25.00  Z=1.48 | **T=-41.00**  **Z=-2.43** |
|  |  |  | p=0.570 | p=0.359 | p=0.496 | p=0.164 | **p=0.012** |
| A 11.25 |  |  |  | T=-33.00  Z=-1.96 | T=-23.00  Z=-1.36 | T=17.00  Z=1.01 | T=-33.00  Z=-1.96 |
|  |  |  |  | p=0.055 | p=0.203 | p=0.359 | p=0.055 |
| A 9.75 |  |  |  |  | T=-3.00  Z=-0.18 | T=27.00  Z=1.60 | **T=-41.00**  **Z=-2.43** |
|  |  |  |  |  | p=0.910 | p=0.129 | **p=0.012** |
| A 8.25 |  |  |  |  |  | **T=37.00**  **Z=2.19** | **T=45.00**  **Z=2.67** |
|  |  |  |  |  |  | **p=0.027** | **p=0.004** |
| A 6.75 |  |  |  |  |  |  | **T=-35.00**  **Z=-2.07** |
|  |  |  |  |  |  |  | **p=0.039** |
| A 5.50 |  |  |  |  |  |  |  |
|  |  |  |  |  |  |  |  |
|  |  |  |  |  |  |  |  |
|  |  |  |  |  |  |  |  |
| **N BrdU+/GFAP+** | A 13.50 | A 10.75 | A 8.75 | A 6.75 | A 4.50 |  |  |
| A 13.50 |  | T=-33.00  Z=-1.96 | **T=-39.00**  **Z=-2.31** | **T=-43.00**  **Z=-2.55** | **T=-45.00**  **Z=-2.67** |  |  |
|  |  | p=0.055 | **p=0.020** | **p=0.008** | **p=0.004** |  |  |
| A 10.75 |  |  | T=-25.00  Z=-1.48 | **T=-45.00**  **Z=-2.67** | **T=-45.00**  **Z=-2.67** |  |  |
|  |  |  | p=0.164 | **p=0.004** | **p=0.004** |  |  |
| A 8.75 |  |  |  | **T=-39.00**  **Z=-2.31** | **T=-45.00**  **Z=-2.67** |  |  |
|  |  |  |  | **p=0.020** | **p=0.004** |  |  |
| A 6.75 |  |  |  |  | **T=-45.00**  **Z=-2.67** |  |  |
|  |  |  |  |  | **p=0.004** |  |  |
| A 4.50 |  |  |  |  |  |  |  |
|  |  |  |  |  |  |  |  |

Table S4

Statistical results of comparisons between DCX+, DCX-tri and DCX-ovo cells/mm^2^ in different telencephalic regions of the pigeon (Wilcoxon Signed Rank Test).

| **DCX total** | HA | HI | HD | M | NF | NI | NC | HF |
| --- | --- | --- | --- | --- | --- | --- | --- | --- |
| HA |  | T=11.00  Z=0.65 | **T=-43.00**  **Z=-2.55** | **T=-45.00**  **Z=-2.67** | **T=-39.00**  **Z=-2.31** | **T=45.00**  **Z=2.67** | **T=-45.00**  **Z=-2.67** | **T=-45.00**  **Z=2.67** |
|  |  | p=0.570 | **p=0.008** | **p=0.004** | **p=0.020** | **p=0.004** | **p=0.004** | **p=0.004** |
| HI |  |  | **T=-45.00**  **Z=-2.67** | **T=-45.00**  **Z=-2.67** | **T=39.00**  **Z=2.31** | **T=-41.00**  **Z=-2.43** | **T=43.00**  **Z=2.55** | **T=39.00**  **Z=2.31** |
|  |  |  | **p=0.004** | **p=0.004** | **p=0.020** | **p=0.012** | **p=0.008** | **p=0.020** |
| HD |  |  |  | T=1.00  Z=0.06 | **T=-45.00**  **Z=-2.67** | **T=-45.00**  **Z=2.67** | **T=-45.00**  **Z=-2.67** | **T=45.00**  **Z=2.67** |
|  |  |  |  | p=1.000 | **p=0.004** | **p=0.004** | **p=0.004** | **p=0.004** |
| M |  |  |  |  | **T=45.00**  **Z=2.67** | **T=-45.00**  **Z=-2.67** | **T=-45.00**  **Z=-2.67** | **T=45.00**  **Z=2.67** |
|  |  |  |  |  | **p=0.004** | **p=0.004** | **p=0.004** | **p=0.004** |
| NF |  |  |  |  |  | T=9.00  Z=0.53 | T=-19.00  Z=-1.13 | **T=-39.00**  **Z=-2.31** |
|  |  |  |  |  |  | p=0.652 | p=0.301 | **p=0.020** |
| NI |  |  |  |  |  |  | **T=-35.00**  **Z=-2.07** | **T=39.00**  **Z=-2.31** |
|  |  |  |  |  |  |  | **p=0.039** | **p=0.020** |
| NC |  |  |  |  |  |  |  | **T=-41.00**  **Z=-2.43** |
|  |  |  |  |  |  |  |  | **p=0.012** |
| HF |  |  |  |  |  |  |  |  |
|  |  |  |  |  |  |  |  |  |
|  |  |  |  |  |  |  |  |  |
| **DCX-tri** | HA | HI | HD | M | NF | NI | NC | HF |
| HA |  | T=21.00  Z=1.24 | **T=-43.00**  **Z=-2.55** | **T=-45.00**  **Z=-2.67** | **T=39.00**  **Z=2.31** | **T=-41.00**  **Z=-2.43** | **T=-45.00**  **Z=-2.67** | T=-7.00  Z=0.42 |
|  |  | p=0.250 | **p=0.008** | **p=0.004** | **p=0.020** | **p=0.012** | **p=0.004** | p=0.734 |
| HI |  |  | **T=-45.00**  **Z=-2.67** | **T=-45.00**  **Z=-2.67** | **T=35.00**  **Z=2.07** | **T=39.00**  **Z=2.31** | **T=39.00**  **Z=2.31** | T=-13.00  Z=-0.77 |
|  |  |  | **p=0.004** | **p=0.004** | **p=0.039** | **p=0.020** | **p=0.020** | p=0.496 |
| HD |  |  |  | T=11.00  Z=0.65 | **T=-45.00**  **Z=-2.67** | **T=45.00**  **Z=2.67** | **T=-45.00**  **Z=-2.67** | **T=35.00**  **Z=2.07** |
|  |  |  |  | p=0.570 | **p=0.004** | **p=0.004** | **p=0.004** | **p=0.039** |
| M |  |  |  |  | **T=-39.00**  **Z=-2.31** | **T=-43.00**  **Z=-2.55** | **T=45.00**  **Z=2.67** | T=-31.00  Z=-1.84 |
|  |  |  |  |  | **p=0.020** | **p=0.008** | **p=0.004** | p=0.074 |
| NF |  |  |  |  |  | T=15.00  Z=0.89 | T=17.00  Z=1.01 | **T=-43.00**  **Z=-2.55** |
|  |  |  |  |  |  | p=0.426 | p=0.359 | **p=0.008** |
| NI |  |  |  |  |  |  | T=-33.00  Z=-1.96 | **T=45.00**  **Z=2.67** |
|  |  |  |  |  |  |  | p=0.055 | **p=0.004** |
| NC |  |  |  |  |  |  |  | **T=-45.00**  **Z=-2.67** |
|  |  |  |  |  |  |  |  | **p=0.004** |
| HF |  |  |  |  |  |  |  |  |
|  |  |  |  |  |  |  |  |  |
|  |  |  |  |  |  |  |  |  |
| **DCX-ovo** | HA | HI | HD | M | NF | NI | NC | HF |
| HA |  | T=-7.00  Z=-0.42 | **T=-45.00**  **Z=-2.67** | **T=-45.00**  **Z=-2.67** | **T=45.00**  **Z=2.67** | **T=-45.00**  **Z=-2.67** | **T=45.00**  **Z=2.67** | **T=45.00**  **Z=2.67** |
|  |  | p=0.734 | **p=0.004** | **p=0.004** | **p=0.004** | **p=0.004** | **p=0.004** | **p=0.004** |
| HI |  |  | **T=-45.00**  **Z=-2.67** | **T=-45.00**  **Z=-2.67** | **T=45.00**  **Z=2.67** | **T=43.00**  **Z=2.55** | **T=-43.00**  **Z=-2.55** | **T=45.00**  **Z=2.67** |
|  |  |  | **p=0.004** | **p=0.004** | **p=0.004** | **p=0.008** | **p=0.008** | **p=0.004** |
| HD |  |  |  | T=-1.00  Z=-0.06 | **T=-45.00**  **Z=-2.67** | **T=-45.00**  **Z=-2.67** | **T=45.00**  **Z=2.67** | **T=45.00**  **Z=2.67** |
|  |  |  |  | p=1.000 | **p=0.004** | **p=0.004** | **p=0.004** | **p=0.004** |
| M |  |  |  |  | **T=45.00**  **Z=2.67** | **T=45.00**  **Z=2.67** | **T=-45.00**  **Z=-2.67** | **T=-45.00**  **Z=-2.67** |
|  |  |  |  |  | **p=0.004** | **p=0.004** | **p=0.004** | **p=0.004** |
| NF |  |  |  |  |  | T=-3.00  Z=-0.18 | T=-29.00  Z=-1.72 | T=-33.00  Z=-1.96 |
|  |  |  |  |  |  | p=0.910 | p=0.098 | p=0.055 |
| NI |  |  |  |  |  |  | **T=43.00**  **Z=-2.55** | **T=-35.00**  **Z=-2.07** |
|  |  |  |  |  |  |  | **p=0.008** | **p=0.039** |
| NC |  |  |  |  |  |  |  | T=31.00  Z=1.84 |
|  |  |  |  |  |  |  |  | p=0.074 |
| HF |  |  |  |  |  |  |  |  |
|  |  |  |  |  |  |  |  |  |

Table S5

Means±S.E. of DCX+, DCX-tri and DCX-ovo cells/mm^2^ of different atlas levels along the anterior-posterior axis in different telencephalic regions of the pigeon.

| HA | DCX all | DCX tri | DCX ovo |
| --- | --- | --- | --- |
| HA |  |  |  |
| A 14.00 | 18.10±1.17 | 13.36±1.15 | 4.74±0.58 |
| A 12.75 | 20.03±1.50 | 14.26±1.54 | 5.77±0.44 |
| A 11.50 | 26.07±4.75 | 17.53±3.72 | 8.54±1.44 |
| A 10.25 | 27.56±4.45 | 18.99±3.63 | 8.57±1.28 |
| A 9.00 | 39.08±11.06 | 24.88±5.22 | 14.19±6.14 |
| A 7.75 | 37.03±5.56 | 24.07±2.34 | 12.96±3.42 |
| HI |  |  |  |
| A 14.00 | 17.05±3.16 | 12.43±2.22 | 4.62±1.36 |
| A 12.75 | 20.82±1.85 | 15.36±1.70 | 5.46±0.29 |
| A 11.50 | 26.73±4.89 | 18.93±4.02 | 7.80±1.32 |
| A 10.25 | 37.87±8.52 | 27.726.88 | 10.15±1.92 |
| HD |  |  |  |
| A 14.00 | 14.37±1.96 | 11.29±1.72 | 3.08±0.42 |
| A 12.75 | 16.82±1.91 | 12.02±1.54 | 4.80±0.61 |
| A 11.50 | 20.42±4.98 | 14.87±3.97 | 5.54±1.10 |
| A 10.25 | 18.57±2.80 | 13.58±2.29 | 4.99±0.79 |
| A 9.00 | 20.68±3.69 | 15.55±2.84 | 5.13±0.93 |
| M |  |  |  |
| A 14.25 | 11.83±1.34 | 9.69±1.04 | 2.88±0.47 |
| A 12.75 | 15.33±1.56 | 11.48±1.37 | 3.92±0.47 |
| A 11.25 | 19.03±3.26 | 14.51±2.71 | 4.59±0.65 |
| A 9.75 | 18.28±2.98 | 13.88±2.52 | 4.60±0.62 |
| A 8.25 | 14.22±1.97 | 11.13±1.55 | 4.24±0.73 |
| A 6.75 | 16.00±3.07 | 11.72±2.42 | 4.28±0.76 |
| N |  |  |  |
| A 13.50 | 108.81±20.20 | 49.78±9.08 | 58.26±11.23 |
| A 10.75 | 107.33±14.70 | 53.42±6.30 | 53.26±8.57 |
| A 8.75 | 102.05±14.92 | 48.00±6.27 | 53.48±8.73 |
| A 6.75 | 73.85±9.22 | 35.21±3.18 | 38.29±6.28 |
| A 4.50 | 89.34±10.33 | 45.47±4.08 | 43.42±7.03 |
| HF |  |  |  |
| A9.50 | 40.64±4.31 | 16.46±1.51 | 24.18±4.87 |
| A8.25 | 41.36±3.57 | 18.48±1.39 | 22.87±3.76 |
| A6.75 | 42.17±4.39 | 18.56±0.97 | 23.58±4.01 |
| A5.50 | 39.90±4.14 | 16.63±1.42 | 23.27±3.81 |
| A4.25 | 25.90±3.50 | 7.90±1.18 | 18.00±2.47 |

Table S6

Statistical results of comparisons between DCX+, DCX-tri and DCX-ovo cells/mm^2^ of different atlas levels and in different telencephalic regions of the pigeon (Wilcoxon Signed Rank Test).

| **HA DCX+** | A 14.00 | A 12.75 | A 11.50 | A 10.25 | A 9.00 | A 7.75 |
| --- | --- | --- | --- | --- | --- | --- |
| A 14.00 |  | T=23.00  Z=1.36 | T=29.00  Z=1.72 | T=31.00  Z=1.84 | **T=39.00**  **Z=2.31** | **T=-45.00**  **Z=-2.67** |
|  |  | p=0.203 | p=0.098 | p=0.074 | **p=0.020** | **p=0.004** |
| A 12.75 |  |  | T=27.00  Z=1.60 | T=33.00  Z=1.96 | **T=43.00**  **Z=2.55** | **T=39.00**  **Z=2.31** |
|  |  |  | p=0.129 | p=0.055 | **p=0.008** | **p=0.020** |
| A 11.50 |  |  |  | T=11.00  Z=0.65 | T=25.00  Z=1.48 | T=23.00  Z=1.36 |
|  |  |  |  | p=0.570 | p=0.164 | p=0.203 |
| A 10.25 |  |  |  |  | T=13.00  Z=0.77 | T=17.00  Z=1.01 |
|  |  |  |  |  | p=0.496 | p=0.359 |
| A 9.00 |  |  |  |  |  | T=-1.00  Z=-0.06 |
|  |  |  |  |  |  | p=1.000 |
| A 7.75 |  |  |  |  |  |  |
|  |  |  |  |  |  |  |

| **HA DCX tri** | A 14.00 | A 12.75 | A 11.50 | A 10.25 | A 9.00 | A 7.75 |
| --- | --- | --- | --- | --- | --- | --- |
| A 14.00 |  | T=-13.00  Z=-0.77 | T=17.00  Z=1.01 | T=23.00  Z=1.36 | **T=37.00**  **Z=2.19** | **T=-41.00**  **Z=-2.43** |
|  |  | p=0.496 | p=0.359 | p=0.203 | **p=0.027** | **p=0.012** |
| A 12.75 |  |  | T=15.00  Z=0.89 | T=27.00  Z=1.60 | **T=41.00**  **Z=2.43** | **T=41.00**  **Z=2.43** |
|  |  |  | p=0.426 | p=0.129 | **p=0.012** | **p=0.012** |
| A 11.50 |  |  |  | T=-15.00  Z=-0.89 | T=23.00  Z=1.36 | T=23.00  Z=1.36 |
|  |  |  |  | p=0.426 | P=0.203 | p=0.203 |
| A 10.25 |  |  |  |  | T=17.00  Z=1.01 | T=15.00  Z=0.89 |
|  |  |  |  |  | p=0.359 | p=0.426 |
| A 9.00 |  |  |  |  |  | T=-3.00  Z=-0.18 |
|  |  |  |  |  |  | p=0.910 |
| A 7.75 |  |  |  |  |  |  |
|  |  |  |  |  |  |  |

| **HA DCX ovo** | A 14.00 | A 12.75 | A 11.50 | A 10.25 | A 9.00 | A 7.75 |
| --- | --- | --- | --- | --- | --- | --- |
| A 14.00 |  | T=27.00  Z=1.60 | T=31.00  Z=1.84 | T=-29.00  Z=-1.72 | **T=39.00**  **Z=2.31** | **T=37.00**  **Z=2.19** |
|  |  | p=0.129 | p=0.074 | p=0.098 | **p=0.020** | **p=0.027** |
| A 12.75 |  |  | T=29.00  Z=1.72 | T=33.00  Z=1.96 | **T=45.00**  **Z=2.67** | **T=37.00**  **Z=2.19** |
|  |  |  | p=0.098 | p=0.055 | **p=0.004** | **P=0.027** |
| A 11.50 |  |  |  | T=1.00  Z=0.06 | T=21.00  Z=1.24 | T=21.00  Z=1.24 |
|  |  |  |  | p=1.000 | p=0.250 | p=0.250 |
| A 10.25 |  |  |  |  | T=11.00  Z=0.65 | T=19.00  Z=1.13 |
|  |  |  |  |  | p=0.570 | p=0.301 |
| A 9.00 |  |  |  |  |  | T=-1.00  Z=-0.06 |
|  |  |  |  |  |  | p=1.000 |
| A 7.75 |  |  |  |  |  |  |
|  |  |  |  |  |  |  |

| **HI DCX tri** | A 14.00 | A 12.75 | A 11.50 | A 10.25 | A 9.00 |  |
| --- | --- | --- | --- | --- | --- | --- |
| A 14.00 |  | T=9.00  Z=0.53 | T=11.00  Z=0.65 | T=27.00  Z=1.60 | Not used |  |
|  |  | p=0.652 | p=0.570 | p=0.129 | (just 4 animals) | |
| A 12.75 |  |  | T=9.00  Z=0.53 | **T=39.00**  **Z=2.31** |  |  |
|  |  |  | p=0.652 | **p=0.020** |  |  |
| A 11.50 |  |  |  | **T=45.00**  **Z=2.67** |  |  |
|  |  |  |  | **p=0.004** |  |  |
| A 10.25 |  |  |  |  |  |  |
|  |  |  |  |  |  |  |
| A 9.00 | Not used (just 4 animals) |  |  |  |  |  |
|  |  |  |  |  |  |  |

| **N DCX ovo** | A 13.50 | A 10.75 | A 8.75 | A 6.75 | A 4.50 |
| --- | --- | --- | --- | --- | --- |
| A 13.50 |  | T=-5.00  Z=-0.30 | T=-13.00  Z=-0.77 | T=-33.00  Z=-1.96 | T=-27.00  Z=-1.60 |
|  |  | p=0.820 | p=0.496 | p=0.055 | p=0.129 |
| A 10.75 |  |  | T=-5.00  Z=-0.30 | **T=-45.00**  **Z=-2.67** | T=-23.00  Z=-1.36 |
|  |  |  | p=0.820 | **p=0.004** | p=0.203 |
| A 8.75 |  |  |  | **T=-45.00**  **Z=-2.67** | T=-29.00  Z=-1.72 |
|  |  |  |  | **p=0.004** | p=0.098 |
| A 6.75 |  |  |  |  | T=29.00  Z=1.72 |
|  |  |  |  |  | p=0.098 |
| A 4.50 |  |  |  |  |  |
|  |  |  |  |  |  |

| **HF DCX all** | A 9.50 | A 8.25 | A 6.75 | A 5.50 | A 4.25 |
| --- | --- | --- | --- | --- | --- |
| A 9.50 |  | T=7.00  Z=0.42 | T=21.00  Z=1.24 | T=-3.00  Z=-0.18 | **T=-43.00**  **Z=-2.55** |
|  |  | p=0.734 | p=0.250 | p=0.910 | **p=0.008** |
| A 8.25 |  |  | T=11.00  Z=0.65 | T=-11.00  Z=-0.65 | **T=-45.00**  **Z=-2.67** |
|  |  |  | p=0.570 | p=0.570 | **p=0.004** |
| A 6.75 |  |  |  | T=-15.00  Z=-0.89 | **T=-45.00**  **Z=-2.67** |
|  |  |  |  | p=0.426 | **p=0.004** |
| A 5.50 |  |  |  |  | **T=-45.00**  **Z=-2.67** |
|  |  |  |  |  | **p=0.004** |
| A 4.25 |  |  |  |  |  |
|  |  |  |  |  |  |

| **HF DCX tri** | A 9.50 | A 8.25 | A 6.75 | A 5.50 | A 4.25 |
| --- | --- | --- | --- | --- | --- |
| A 9.50 |  | T=27.00  Z=1.60 | T=23.00  Z=1.36 | T=13.00  Z=0.77 | **T=-41.00**  **Z=-2.43** |
|  |  | p=0.129 | p=203 | p=0.496 | **p=0.012** |
| A 8.25 |  |  | T=9.00  Z=0.53 | T=-11.00  Z=0.65 | **T=-43.00**  **Z=-2.55** |
|  |  |  | p=0.652 | p=0.570 | **p=0.008** |
| A 6.75 |  |  |  | T=-23.00  Z=1.36 | **T=-43.00**  **Z=-2.55** |
|  |  |  |  | p=0.203 | **p=0.008** |
| A 5.50 |  |  |  |  | **T=-43.00**  **Z=-2.55** |
|  |  |  |  |  | **p=0.008** |
| A 4.25 |  |  |  |  |  |
|  |  |  |  |  |  |
